# A Bacterial Effector Hijacks NBR1 to Modulate Both Autophagy and Ubiquitination‐Mediated Degradation That Promotes Bacterial Infection

**DOI:** 10.1111/pbi.70509

**Published:** 2025-12-17

**Authors:** Yaqian Shi, Fang Fang, Xuejin Cui, Hongwei Shi, Zaiyu Yang, Xueyi Li, Changyong Zhou, Xuefeng Wang

**Affiliations:** ^1^ National Citrus Engineering Research Center, Citrus Research Institute Southwest University Chongqing China; ^2^ Integrative Science Center of Germplasm Creation in Western China (CHONGQING) Science City Southwest University Chongqing China; ^3^ Cucurbit Vegetable Innovation Team, Institute of Vegetables and Flowers Chongqing Academy of Agricultural Sciences Chongqing China

**Keywords:** autophagic receptor, autophagy, *Candidatus* Liberibacter asiaticus, E3 ligase, huanglongbing, UPS

## Abstract

Autophagy and the ubiquitin/26S proteasome system (UPS) play critical roles in the immune defence of the host against pathogen invasion. As a countermeasure, pathogens deploy effector proteins to subvert or hijack autophagy and UPS processes. However, it is unclear whether and how a single pathogen effector coordinately modulates both proteolytic systems. Here, we identified a RING finger E3 ligase of *Citrus sinensis* , CsRHY1A, that directly interacts with SDE4405, an effector protein from *Candidatus* Liberibacter asiaticus (*C*Las), the causal agent of citrus Huanglongbing (HLB). CsRHY1A ubiquitinated SDE4405 at Lys87 and Lys92, causing SDE4405 degradation via the 26S proteasome. Furthermore, SDE4405 targeted the ubiquitin‐associated (UBA) domain of the autophagic receptor NEIGHBOR OF BRCA1 (CsNBR1) and competitively disrupted CsRHY1A‐mediated degradation by decreasing the ubiquitination of SDE4405. Lys87 and Lys92 of SDE4405 were required for its interactions with CsRHY1A and CsNBR1 and were essential for CsNBR1‐dependent stabilisation of SDE4405. SDE4405 also inhibited the binding of CsNBR1 to CsATG8s, suppressing CsNBR1‐mediated selective autophagic degradation of *C*Las effector protein SDE1. These findings reveal the sophisticated strategy of bacteria to counteract both autophagy and proteasome‐dependent degradation, providing opportunities for developing HLB‐resistant citrus varieties.

## 1 Introduction

The two main cellular proteolytic machineries, the ubiquitin/26S proteasome system (UPS) and autophagy, act in parallel and differentially under changing pathophysiological conditions, maintaining homeostasis and quality control of the proteome and organelles (Pohl and Dikic 2019). UPS regulates small, short‐lived proteins, whereas autophagy is the degradative route for large, heterogeneous cytoplasmic materials, such as protein aggregates, organelles and invading bacteria whose sizes exceed the spatial capacity of proteasomes (Cohen‐Kaplan, Livneh, Avni, Fabre, et al. 2016; Pankiv et al. 2007). A common regulatory step shared by the two systems is the signalling of their targets for destruction via the covalent attachment of ubiquitin (Ub), a conserved 76 amino acid protein present in all eukaryotes (Cohen‐Kaplan, Livneh, Avni, Cohen‐Rosenzweig, and Ciechanover 2016; Finley 2009).

Ubiquitin‐activating enzyme E1, ubiquitin‐conjugating enzyme E2, and ubiquitin ligase E3 coordinate ubiquitination. E3 ligases, including the RING‐finger E3 ligases, which harbour a characteristic set of eight conserved Cys and His residues, are closely associated with the plant immunity response to pathogen invasion (Fu et al. 2022; Wu et al. 2024; Yu et al. 2013; Zhong et al. 2018). For example, C3H2C3‐type RING E3 ligase NtRFP1 in tobacco mediates the ubiquitination and degradation of a geminivirus‐encoded βC1 protein, decreasing disease symptoms (Shen et al. 2016). The C3H2C3‐type RING E3 ligase NbHRD1a triggers the degradation of beet necrotic yellow vein virus‐encoded triple gene block movement proteins through the 26S proteasome, enhancing plant antiviral immunity (Guo et al. 2025). Similarly, C3HC4‐type RING E3 ligase OsRGLG5 targets rice blast fungus effector AvrPi9, positively regulating basal resistance to the rice blast fungus (Liu et al. 2023).

Selective autophagy is a key regulator of innate immunity that degrades pathogen components and/or host factors that are essential for infection (Marshall and Vierstra 2018; Tong et al. 2023). In this process, NEIGHBOR OF BRCA1 (NBR1), a core autophagic receptor, binds ubiquitinated substrates at its Ub‐associated (UBA) domain and delivers them to developing autophagosomes for degradation (Svenning et al. 2011; Zhou et al. 2021). *Arabidopsis* NBR1 (AtNBR1) directly targets the capsid protein and particles of cauliflower mosaic virus for degradation (Hafrén et al. 2017). In addition, NBR1‐mediated selective autophagy suppresses turnip mosaic virus infection by targeting the viral RNA‐silencing suppressor HC‐Pro (Hafrén et al. 2018).

Pathogens have evolved strategies to manipulate the plant proteasome and autophagy pathways to their own benefit, overcoming these UPS and autophagy‐mediated defences. For example, the rice stripe virus‐encoded NS3 protein interferes with the microtubule‐associated E3‐ligase (MEL)‐serine hydroxymethyltransferase 1 (SHMT1) module‐mediated defence response by competitively binding to the MEL substrate recognition site, thereby inhibiting MEL from interacting with and ubiquitinating SHMT1 (Wang, Fu, et al. 2023). The C2 protein of tomato yellow leaf curl virus suppresses plant defences against insect vectors by interacting with ubiquitin‐40S ribosomal protein S27a (RPS27A), subverting ubiquitination (Li et al. 2019). *Phytophthora infestans* PexRD54 competes with NBR1/Joka2 to bind to host ATG8CL, interfering with the positive effect of Joka2 on pathogen defence (Dagdas et al. 2016). NBR1 interacts with the βC1 protein encoded by tomato yellow leaf curl China betasatellite, forming cytoplasmic granules that evade degradation by the E3 ligase NbRFP1‐mediated ubiquitination pathway and thus promoting virus infection (Zhou et al. 2021). The XopL effector of *Xanthomonas campestris* pv. *vesicatoria* (*Xcv*) is targeted for degradation by NBR1/Joka2. Ubiquitinated XopL then degrades autophagy component SH3P2 in a proteasome‐dependent manner, suppressing autophagic degradation (Leong et al. 2022). However, the possible combined manipulation of both UPS and autophagy by a single pathogen effector has not previously been considered.

Citrus huanglongbing (HLB), the most devastating citrus disease worldwide, is caused by the phloem‐colonising bacterium *Candidatus* Liberibacter asiaticus (*C*Las) (Bove 2006). Genome analysis has shown that *C*Las possesses a complete type I secretion system and Sec‐dependent secretory machinery (Duan et al. 2009; Wang and Trivedi 2013). Sec‐delivered effectors (SDEs) are released into the phloem sieve cells and neighbouring companion cells and move through plasmodesmata (Pagliaccia et al. 2017; Pitino et al. 2016; Prasad et al. 2016). Recently, two *C*Las effectors, SDE4405 and SDE3, have been shown to directly or indirectly regulate autophagy‐related ATG8 proteins, facilitating bacterial infection (Shi, Yang, et al. 2023; Shi, Gong, et al. 2023). *C*Las effector SDE5 enhances the interaction between plant U‐box (PUB) type E3 ligase PUB21 and transcription factor MYC2, thereby promoting PUB21‐mediated proteolysis of MYC2 (Zhao et al. 2025). In this study, we focused on the SDE4405 effector to reveal the mechanism by which SDE4405 escapes host defences and promotes HLB infection (Shi, Yang, et al. 2023). We found that SDE4405 was ubiquitinated by the E3 ligase CsRHY1A, a positive regulator of the defence response against *C*Las, and degraded via the 26S proteasome pathway. SDE4405 then targeted the autophagic receptor CsNBR1 and competitively disrupted CsRHY1A‐mediated degradation. SDE4405 also inhibited CsNBR1‐mediated selective autophagic degradation of *C*Las effector protein SDE1. Thus, these results show that SDE4405 modulates both proteasomal and autophagic degradation pathways, revealing the unprecedented virulence mechanism of this *C*Las effector.

## 2 Results

### 2.1 SDE4405 Interacts With and Is Ubiquitinated by RING‐Type E3 Ligase RHY1A

To explore the regulation of SDE4405 protein abundance, Myc‐tagged SDE4405 was transiently expressed in *Nicotiana benthamiana* leaves. As protein abundance is tightly controlled by its biosynthesis and degradation, we determined the consequences of translational inhibition using cycloheximide (CHX) treatment. Myc‐SDE4405 protein accumulation rapidly decreased 3–9 h after CHX exposure, which was blocked by concomitant incubation with proteasome inhibitor MG132 (Figure S1a–c). In contrast, treatment with E64d, an inhibitor of aspartic and cysteine proteases, did not significantly affect SDE4405 protein degradation (Figure S1a–c). Using a cell‐free degradation method, we found that the half‐life of recombinant His‐SUMO‐SDE4405 was shortened when incubated with crude protein extracts from citrus leaves, as the His‐SUMO‐SDE4405 content in the extracts gradually decreased as the incubation time was extended. However, MG132 treatment prevented His‐SUMO‐SDE4405 degradation (Figure S1d). In addition, we transiently co‐expressed Myc‐SDE4405 with Flag‐tagged ubiquitin in *N. benthamiana* leaves and determined the ubiquitination of the immunoprecipitated SDE4405 protein by immunoblotting (Figure S1e). Collectively, these results demonstrate that SDE4405 is ubiquitinated and degraded via the 26S proteasome.

**FIGURE 1:**
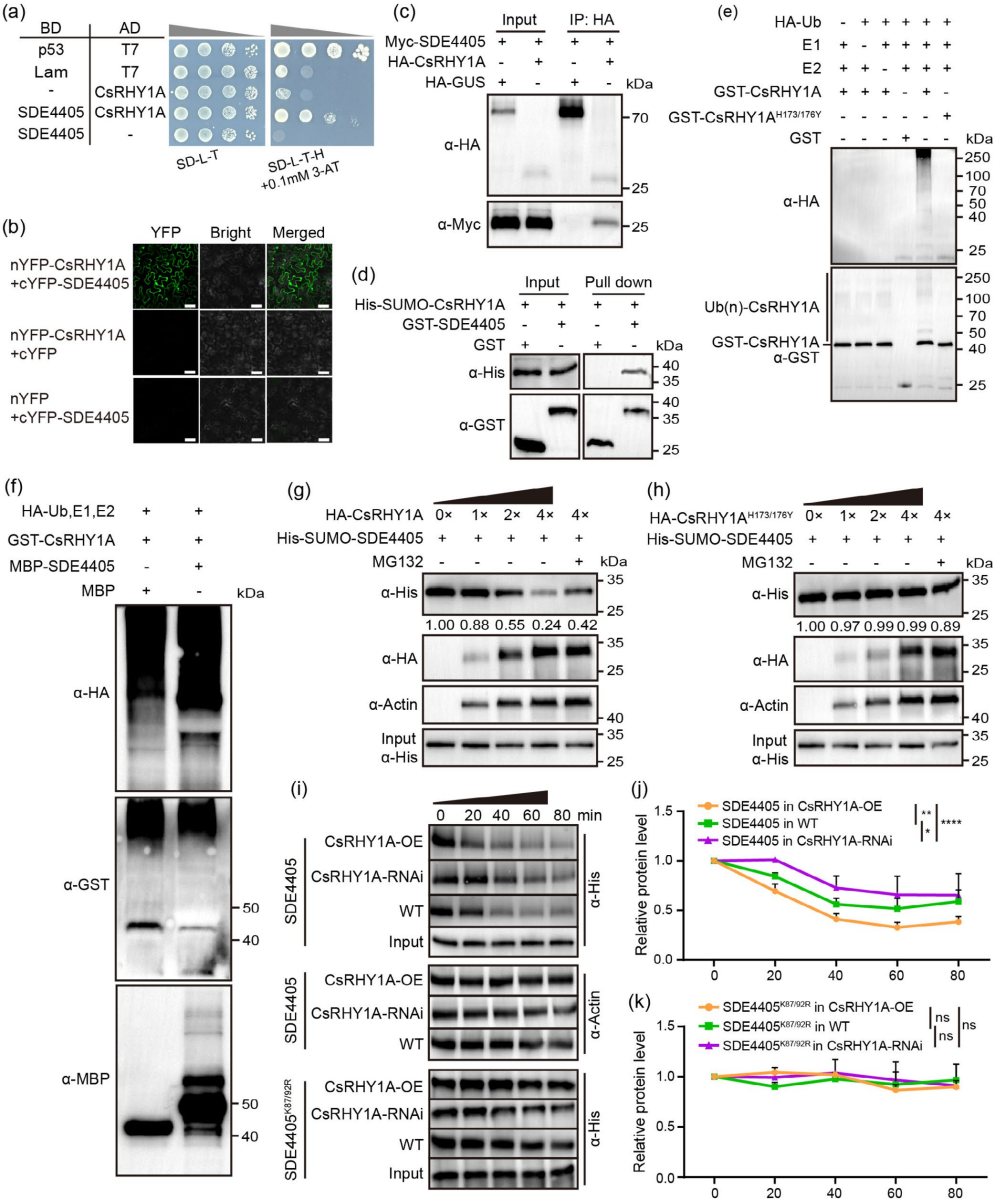
CsRHY1A targets SDE4405 for ubiquitination and degradation. (a) Yeast two‐hybrid (Y2H) analysis confirming the specific interaction between SDE4405 and CsRHY1A. (b) Bimolecular fluorescence complementation (BiFC) assays demonstrating the interaction between SDE4405 and CsRHY1A in *Nicotiana benthamiana* leaves. Confocal imaging was performed at 48 h post‐inoculation (hpi). Scale bar = 50 μm. (c) Co‐immunoprecipitation (Co‐IP) analysis confirming the in vivo interaction between SDE4405 and CsRHY1A. (d) GST pull‐down assays examining the in vitro interaction between His‐SUMO‐CsRHY1A and GST‐SDE4405. GST‐SDE4405 or GST (negative control) was incubated with His‐SUMO‐CsRHY1A. (e) In vitro self‐ubiquitination assays of CsRHY1A and its mutant CsRHY1A^H173/176Y^. GST‐CsRHY1A, GST‐CsRHY1A^H173/176Y^, or GST (negative control) were incubated with E1, E2, and HA‐ubiquitin (Ub) to assess E3 ligase activity. (f) In vitro ubiquitination assays demonstrating SDE4405 ubiquitination by CsRHY1A. MBP as a negative control. (g–k) Cell‐free degradation assays illustrating CsRHY1A‐mediated SDE4405 degradation. SDE4405 protein abundance in *N. benthamiana* leaves expressing HA‐CsRHY1A or HA‐CsRHY1A^H173/176Y^ proteins incubated with His‐SUMO‐SDE4405 recombination proteins with or without MG132 (g, h). Degradation of His‐SUMO‐SDE4405 and His‐SUMO‐SDE4405^K87/92R^ in cell‐free assays from wild‐type (WT), *CsRHY1A* overexpression (*CsRHY1A*‐OE), and *CsRHY1A* RNA‐interfering (*CsRHY1A*‐RNAi) protein extracts after 0, 20, 40, 60, and 80 min (i–k). MG132 was used to stop the degradation in samples collected at the pointed time. Data represent mean ± SD of three biological replicates (*p < 0.05, **p < 0.01, ****p < 0.0001, two‐way ANOVA).

During ubiquitin‐dependent protein degradation, E3 ligases are the primary regulators of substrate specificity. Based on the candidate proteins initially identified in our preliminary yeast two‐hybrid (Y2H) screening (Shi, Yang, et al. 2023), we confirmed through Y2H assays that the E3 ubiquitin ligase CsRHY1A (GenBank accession: XM_006473879.4) interacted with SDE4405 (Figure 1a). The interaction was further confirmed using an in planta bimolecular fluorescence complementation (BiFC) assay (Figure 1b), an in vivo Co‐immunoprecipitation (Co‐IP) assay (Figure 1c), and an in vitro pull‐down assay (Figure 1d). Given that SDE4405 is also degraded by the 26S proteasome in *N. benthamiana* (Figure S1a–c), we found that it also interacted with NbRHY1A (Niben101Scf12672g00008.1), the homologue of CsRHY1A (Figure S2a,b).

Previous studies have established that RING finger‐containing proteins comprise a major E3 ligase subclass that regulates substrate ubiquitination through direct interactions with E2 and target proteins (Meng et al. 2023; Wu et al. 2024). The C‐terminus of CsRHY1A contains a C3‐H2‐C3‐type RING‐finger domain (149–195 amino acids) with conserved cysteine (Cys) and histidine (His) residues (Figure S3a). The RING domain of RHY1A is highly conserved in the *Citrus* genus (Figure S3b). An in vitro E3 ligase assay was performed to determine whether CsRHY1A has E3 ligase activity; the results showed that CsRHY1A is a functional E3 ligase (Figure 1e). The RING motif in RING‐type E3 ligase proteins is required for E3 ligase activity (Liu et al. 2023; Wu et al. 2024). His 173 and 176 were replaced with tyrosine (CsRHY1A^H173/176Y^) to disrupt the RING domain (Figure S3c), and mutated CsRHY1A did not exhibit ubiquitin ligase activity (Figure 1e), demonstrating that the RING finger domain is indispensable for E3 ligase activity in CsRHY1A. We then performed a ubiquitination assay and showed that CsRHY1A efficiently polyubiquitinated SDE4405 in vitro (Figure 1f).

We performed cell‐free degradation assays to determine whether CsRHY1A promotes SDE4405 degradation. His‐SUMO‐SDE4405 was incubated with HA‐CsRHY1A or HA‐CsRHY1A^H173/176Y^ protein extracts from *N. benthamiana* leaves with or without MG132. The SDE4405 protein level gradually decreased in a CsRHY1A‐dependent manner, and this effect was efficiently inhibited by MG132 (Figure 1g). However, SDE4405 degradation was partially reduced after incubation with HA‐CsRHY1A^H173/176Y^ protein extracts in comparison to wild‐type (WT) HA‐CsRHY1A (Figure 1h). In addition, in vivo protein degradation assays were performed with agroinfiltration in *N. benthamiana*. Quantitative immunoblot analysis revealed that SDE4405 protein accumulation levels were reduced in leaves co‐expressing HA‐CsRHY1A compared to those expressing HA‐GUS (Figure S2c). The pretreatment of leaves with MG132 significantly inhibited Flag‐SDE4405 degradation by HA‐CsRHY1A (Figure S2d). NbRHY1A also promoted the degradation of SDE4405 via the 26S proteasome (Figure S2e,f).

Total protein extracts from WT, *CsRHY1A* overexpression (*CsRHY1A*‐OE), and *CsRHY1A* RNA‐interfering (*CsRHY1A*‐RNAi) citrus leaves were incubated with purified His‐SUMO‐SDE4405 in cell‐free degradation assays to further determine whether SDE4405 is specifically degraded by CsRHY1A via the ubiquitin‐dependent 26S proteasome system in citrus. We found that incubating SDE4405 with protein extracts from *CsRHY1A*‐OE plants resulted in more rapid degradation, while SDE4405 was slowly degraded when incubated with *CsRHY1A*‐RNAi plant protein extracts (Figure 1i–k). Similar results were obtained when using recombinant protein GST‐SDE4405 as a substrate for WT, *CsRHY1A*‐OE, and ‐RNAi fusion proteins (Figure S2g). Collectively, these results demonstrate that CsRHY1A is responsible for SDE4405 protein degradation.

To identify ubiquitinated residues in the SDE4405 protein, we performed liquid chromatography–tandem mass spectrometry (LC–MS/MS) analysis after transiently expressing and immunoprecipitating SDE4405‐GFP from *N. benthamiana* leaves. MS/MS analysis identified two distinct ubiquitin‐conjugated tryptic peptides corresponding to the Lys87 and Lys92 residues in SDE4405 (Figure S4a). To reveal the function of these Lys sites in SDE4405 ubiquitination by RHY1A, we mutagenized Lys to Arg and found that the ubiquitination of SDE4405 was diminished, especially in SDE4405^K87/92R^, suggesting that the two Lys sites are critical for CsRHY1A‐mediated SDE4405 ubiquitination (Figure S4b). Structural‐guided mutagenesis of the conserved ubiquitination acceptor sites (K87/92R) abolished CsRHY1A‐SDE4405 complex formation (Figure S4c). We also examined the degradation of recombinant His‐SUMO‐SDE4405^K87/92R^ and GST‐SDE4405^K87/92R^ incubated with total protein extracted from WT, *CsRHY1A*‐OE, and ‐RNAi citrus leaves in a cell‐free system and found that recombinant His‐SUMO‐SDE4405^K87/92R^ and GST‐SDE4405^K87/92R^ were stable when incubated with these extracts (Figure 1i–k and Figure S2g). These results indicate that ubiquitination at the Lys87 and Lys92 sites determines the stability of the SDE4405 protein.

### 2.2 CsRHY1A Positively Regulates the Resistance of Citrus to *C*Las

**FIGURE 2:**
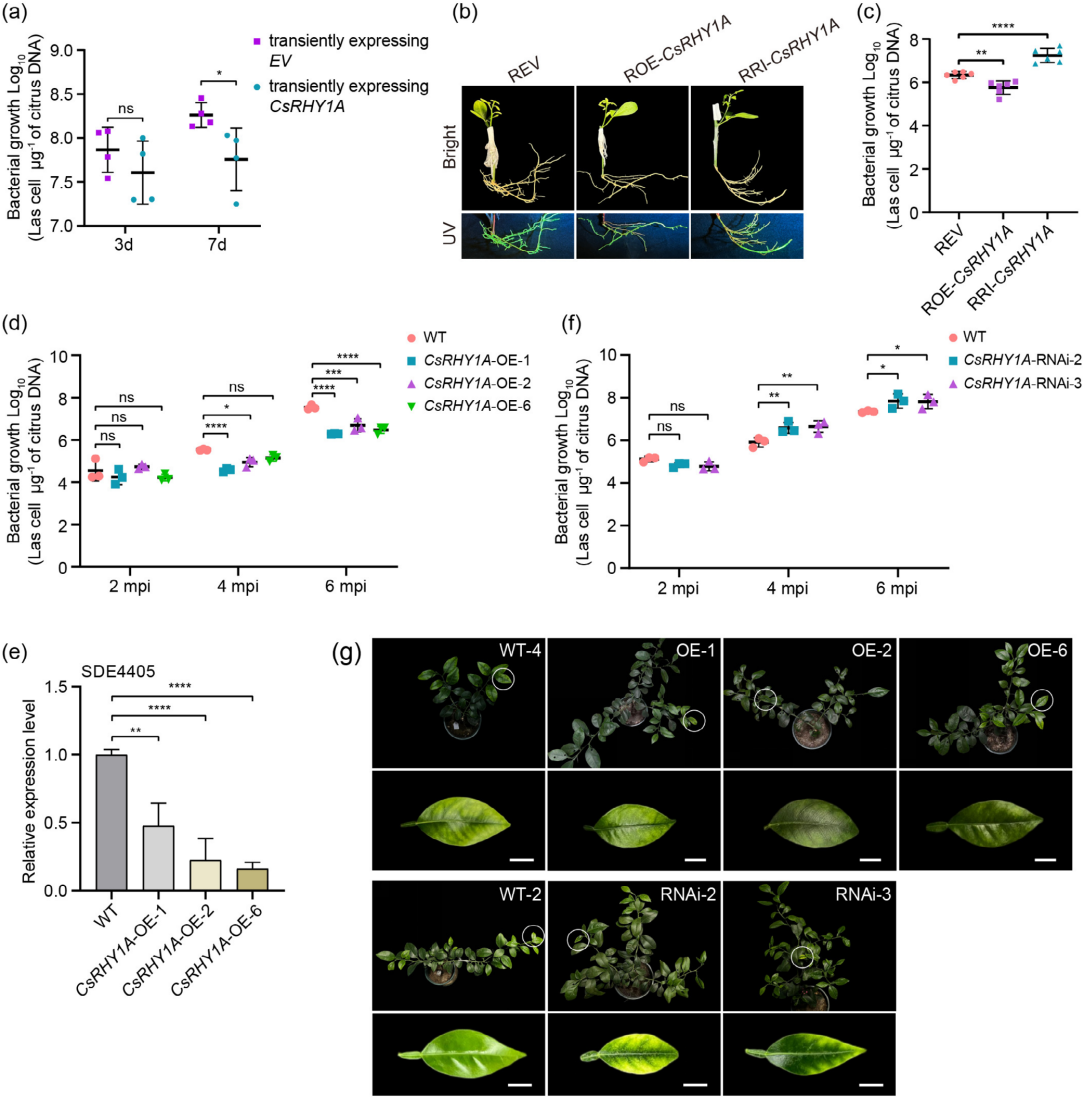
CsRHY1A positively regulates citrus disease resistance to *Candidatus* Liberibacter asiaticus (*C*Las). (a) *C*Las titers in *Citrus sinensis* leaves transiently expressing CsRHY1A or empty vector (EV) after *C*Las infection. Values are means ± SD (*n* = 4) (*p < 0.05, Student's *t*‐test). (b) Green fluorescent protein (GFP) signals visualised under UV light in EV (REV), overexpressing *CsRHY1A* (ROE‐*CsRHY1A*), and RNA interfering *CsRHY1A* (RRI‐*CsRHY1A*) citrus root tissues. (c) *C*Las titers in REV, ROE‐*CsRHY1A* and RRI‐*CsRHY1A* citrus hairy roots at 25 dpi via grafting with buds from *C*Las‐infected citrus plants. Values are means ± SD of six biological replicates (**p < 0.01, ****p < 0.0001, one‐way ANOVA). (d) Quantitative analysis of *C*Las growth at 2, 4 and 6 months post‐inoculation (mpi) in *CsRHY1A*‐OE and WT citrus plants. Values are means ± SD of three biological replicates (ns indicates no significant differences; *p < 0.05, ***p < 0.001, ****p < 0.0001, two‐way ANONA). (e) Relative mRNA levels of SDE4405 in WT and *CsRHY1A*‐OE citrus leaves at 6 mpi. Values are means ± SD of three biological replicates (**p < 0.01, ****p < 0.0001, one‐way ANOVA). (f) *C*Las titers at 2, 4 and 6 mpi in *CsRHY1A*‐RNAi and WT citrus plants. Values are means ± SD of three biological replicates (*p < 0.05, **p < 0.01, one‐way ANOVA). (g) Leave symptoms of WT and *CsRHY1A* transgenic citrus after *C*Las infection for 9 months. The white circles indicate the areas that are enlarged on the panels below. Scale bar = 1 cm.

To determine whether and how CsRHY1A functions in citrus HLB resistance, we transiently expressed CsRHY1A in *C*Las‐infected citrus leaves and found that CsRHY1A upregulation was correlated with a decrease in the titre of *C*Las bacteria (Figure 2a). To further confirm the positive effect of CsRHY1A on disease resistance, 35S:CsRHY1A overexpression and RNAi pNmGFPer‐CsRHY1A constructs were transformed into Citrange, generating ROE‐*CsRHY1A* and RRI‐*CsRHY1A* transgenic hairy roots (Figure 2b and Figure S5). At 25 days after graft inoculation, the *C*Las titers were significantly increased in RRI‐*CsRHY1A* hairy roots, whereas they were reduced in ROE‐*CsRHY1A* hairy roots (Figure 2c). We generated stable transgenic citrus plants in *Citrus sinensis* variety ‘WanJincheng’ to further determine whether CsRHY1A enhanced resistance to *C*Las (Figure S6). Citrus plants were graft‐inoculated using stems from diseased trees. A significant decrease in the *C*Las titre was detected in *CsRHY1A*‐OE compared to WT at 4 and 6 months post‐inoculation (mpi) (Figure 2d). RT‐qPCR analysis showed that SDE4405 transcription in *C*Las‐infected *CsRHY1A*‐OE lines was significantly lower than in WT at 6 mpi (Figure 2e). *CsRHY1A*‐RNAi lines displayed significantly higher *C*Las titers at 4 and 6 mpi compared to WT, with the difference diminishing at 6 mpi (Figure 2f). Ultimately, the most pronounced *C*Las‐associated zinc deficiency and yellowing symptoms were observed in *CsRHY1A*‐RNAi plants at 9 mpi, whereas *CsRHY1A*‐OE lines were largely free of these symptoms (Figure 2g). These results indicate that CsRHY1A confers HLB resistance in citrus.

### 2.3 CsNBR1 Interacts With and Stabilises SDE4405

**FIGURE 3:**
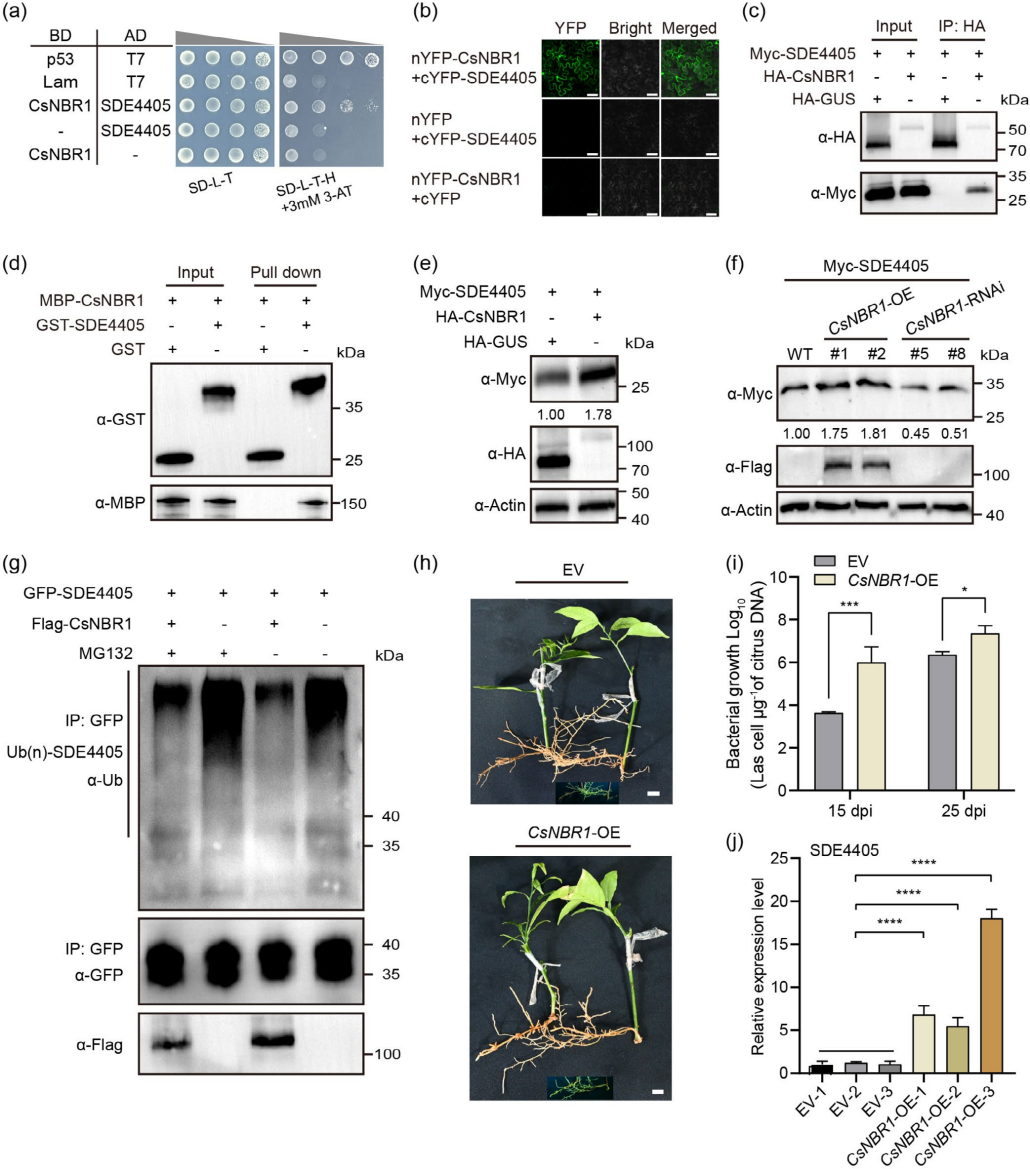
CsNBR1 enhances stability of SDE4405 by suppressing its ubiquitination. (a) Y2H assays demonstrating the interaction between SDE4405 and full‐length CsNBR1. (b) BiFC analysis of SDE4405‐CsNBR1 interactions. Scale bar = 50 μm. (c) Co‐IP analysis confirming the in vivo interaction between SDE4405 and CsNBR1. (d) GST pull‐down detecting the in vitro interactions between SDE4405 and CsNBR1. GST‐SDE4405 or GST was incubated with MBP‐CsNBR1. (e) SDE4405 protein stability assay in split *Nicotiana benthamiana* leaves. Myc‐SDE4405 was co‐expressed with HA‐GUS in one‐half leaf of *N. benthamiana* leave and with CsNBR1 in another half of the same leaf. (f) SDE4405 abundance in stable *CsNBR1* transgenic citrus plants. Transient expression of Myc‐SDE4405 was analysed in WT, Flag‐*CsNBR1*‐OE, and *CsNBR1*‐RNAi plants, with protein accumulation levels detected by immunoblotting. (g) Ubiquitination levels of SDE4405 with or without CsNBR1 and MG132. SDE4405‐GFP was expressed alone or co‐expressed with Flag‐CsNBR1 in *N. benthamiana* leaves for 36 h, followed by MG132 or Dimethyl Sulfoxide (DMSO) for 12 h. Ubiquitination was detected by immunoblotting with an anti‐Ub antibody. (h) GFP signals visualised under UV light in citrus root tissues (Scale bar = 2 cm). (i) *C*Las titers in *CsNBR1*‐OE citrus hairy roots at 15 and 25 dpi graft‐inoculated using branches from *C*Las‐infected citrus plants. Values are means ± SD of three biological replicates (**p < 0.01, Student's *t* test). (j) Relative mRNA levels of SDE4405 in *CsNBR1*‐OE citrus hairy roots at 15 dpi. *CsActin* was used as a control gene for normalisation. Values are means ± SD of three biological replicates (****p < 0.0001, one‐way ANOVA).

Previous studies have demonstrated that transgenic *SDE4405* citrus plants show enhanced *C*Las colonisation (Shi, Yang, et al. 2023). We hypothesised that SDE4405 interacts with other host targets to maintain its stability and act as a virulence effector. Therefore, we also conducted a Y2H assay, using the SDE4405 protein without the signal peptide as bait and a cDNA library prepared from *C. sinensis* leaves. From this screening, 10 *C. sinensis* proteins were identified as candidate SDE4405‐interacting proteins (Table S1). NEIGHBOR OF BRCA1 (CsNBR1) (GenBank accession: XP_006476885.2) was identified by Y2H assay (Figure 3a). The BiFC assays revealed that SDE4405 interacted with CsNBR1 (Figure 3b). We then confirmed the interaction between SDE4405 and full‐length CsNBR1 in planta in the Co‐IP assay using the tobacco transient expression system (Figure 3c). In addition, we found that SDE4405 directly interacted with CsNBR1 in vitro (Figure 3d). We expressed SDE4405 and CsNBR1 in *N. benthamiana* leaves to determine whether they were co‐localised. When SDE4405‐GFP and CsNBR1‐GFP were separately expressed, SDE4405‐GFP localised to the cytoplasm and nucleus, whereas CsNBR1‐GFP revealed green fluorescence in cytoplasmic granules of the cytoplasm (Figure S7a). Co‐expressing SDE4405‐BFP and CsNBR1‐GFP resulted in green fluorescent cytoplasmic granules. The SDE4405‐CsNBR1 interaction complex co‐localised with endoplasmic reticulum markers (Figure S7b).

CsNBR1 contained four conserved domains: an N‐terminal PB1 domain, a ZZ‐like zinc finger domain, a NBR1 domain, and a C‐terminal ubiquitin‐associated (UBA) domain (Figure S8a). To determine whether the UBA domain of CsNBR1 is essential for mediating its binding with SDE4405, we tested the interaction between SDE4405 and CsNBR1^UBA^ (the UBA domain of CsNBR1) or CsNBR1^ΔUBA^ (CsNBR1 lacking the UBA domain) using a Y2H assay. In yeast, CsNBR1 physically interacted with SDE4405 through the UBA domain (Figure S8b). BiFC and Co‐IP assays further confirmed that the UBA domain was required for the interaction between SDE4405 and CsNBR1 (Figure S8c,d). As the UBA domain of NBR1 mediates selective binding to ubiquitin‐modified substrates (Xiang et al. 2024; Zhou et al. 2021), these findings indicate that CsNBR1 recruits ubiquitinated SDE4405 through its UBA domain.

To investigate the regulatory role of NBR1 in SDE4405 stability, we compared Myc‐SDE4405 protein accumulation in *N. benthamiana* leaves co‐expressing either HA‐CsNBR1 or HA‐GUS control. Immunoblot analysis revealed that HA‐CsNBR1 co‐expression significantly enhanced Myc‐SDE4405 accumulation compared to the GUS control (Figure 3e). SDE4405 interacted with NbNBR1, and their interaction occurred within the UBA domain (Figure S9a–c). SDE4405 was then transiently expressed in *NbNBR1*‐OE and WT *N. benthamiana* leaves. Immunoblot analysis showed that SDE4405 protein accumulation was higher in *NbNBR1*‐OE than in WT (Figure S9d–g). Conversely, targeted silencing of the endogenous NbNBR1 homologue via TRV2‐mediated virus‐induced gene silencing (VIGS) resulted in a significant reduction in the Myc‐SDE4405 protein level compared to the TRV2‐GFP control (Figure S9h–j). In addition, transient SDE4405 expression revealed higher protein abundance in *CsNBR1*‐OE citrus plants than in WT, with the lowest levels detected in *CsNBR1*‐RNAi citrus plants (Figure 3f). These results suggest that NBR1 enhances SDE4405 protein stability. We investigated the effect of CsNBR1^UBA^ on SDE4405 protein stability using a cell‐free degradation assay and found that it stabilised the SDE4405 protein, similar to the effect of MG132 (Figure S8e,f). We extracted total proteins from *N. benthamiana* leaves expressing SDE4405‐GFP or SDE4405‐GFP/Flag‐CsNBR1 with or without MG132 treatment and incubated them with Flag magnetic beads to determine whether CsNBR1 affects SDE4405 ubiquitination via the 26S proteasome. CsNBR1 decreased SDE4405 ubiquitination even under MG132 treatment (Figure 3g). SDE4405 ubiquitination was also lower in *NbNBR1*‐OE than in WT (Figure S9k). These results suggest that CsNBR1 decreases SDE4405 ubiquitination and thus maintains its protein stability.

*CsNBR1*‐OE hairy roots were generated using *C*Las‐free *SDE4405*‐OE citrus branches to evaluate their resistance against HLB (Figure 3h and Figure S10). The *CsNBR1*‐OE hairy roots were graft‐inoculated with *C*Las. At 15 dpi, the SDE4405 transcript level and *CLas* titre were significantly increased in *CsNBR1*‐OE hairy roots compared to the empty vector (Figure 3i,j). At 25 dpi, the difference in *C*Las titre between the transgenic hairy roots and the empty vector controls gradually diminished (Figure 3i). Therefore, these results indicate that CsNBR1 facilitates the early infection of *C*Las in *SDE4405*‐OE citrus.

### 2.4 Lys87/Lys92 Are the Key Sites for NBR1 to Stabilise SDE4405

**FIGURE 4:**
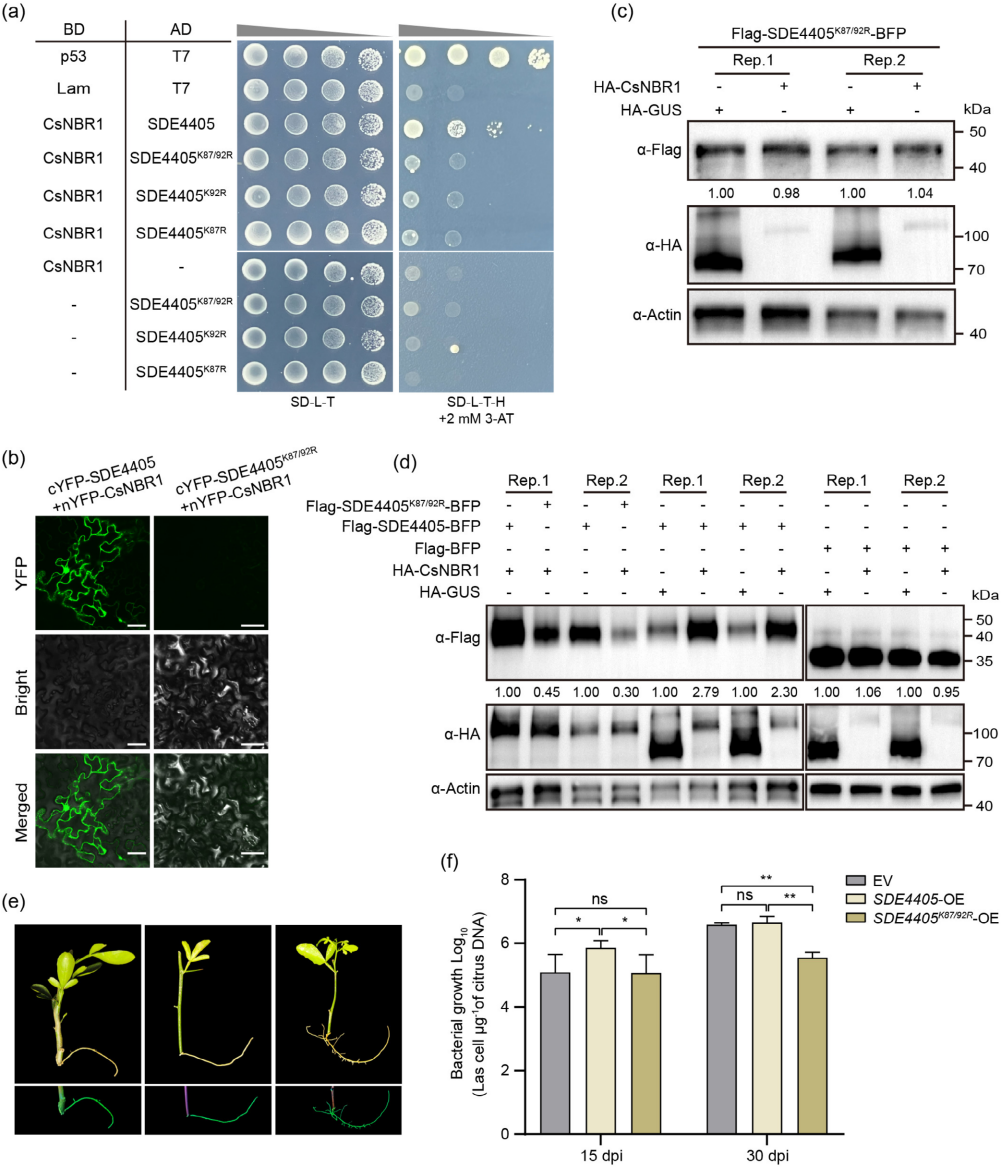
CsNBR1 does not affect SDE4405^K87/92R^ protein stability. (a) Y2H assays analysing the interaction between CsNBR1 and SDE4405^K87/92R^. (b) BiFC assays examining interactions between SDE4405^K87/92R^ and CsNBR1. Scale bar = 50 μm. (c, d) Immunoblot analysis of SDE4405 and SDE4405^K87/92R^ protein levels in *N. benthamiana* leaves co‐expressing HA‐CsNBR1. The protein accumulation levels of Flag‐SDE4405‐BFP (c) or Flag‐SDE4405^K87/92R^‐BFP (c, d) were detected by immunoblotting with an anti‐Flag antibody at 48 hpi. The experiments were repeated at least three times with similar results. (e) GFP signals visualised under UV light in EV, *SDE4405*‐OE, and *SDE4405* ^*K87/92R*^‐OE citrus root tissues. (f) *C*Las titers in *SDE4405*‐OE and *SDE4405* ^*K87/92R*^‐OE citrus hairy roots at 15 and 30 dpi. Values are means ± SD of three biological replicates (*p < 0.05, **p < 0.01, two‐way ANOVA).

Our identification of Lys87 and Lys92 as ubiquitination sites in SDE4405 (Figure S4a) supports the hypothesis that these residues may act as molecular anchors for CsNBR1 binding. Y2H and BiFC assays revealed that mutations at Lys87 and Lys92 of SDE4405 abolished the interaction with CsNBR1 (Figure 4a,b), demonstrating that Lys87 and Lys92 residues are structurally and functionally critical for mediating SDE4405‐CsNBR1 complex formation. Furthermore, our findings revealed that Lys87 and Lys92 were not critical residues for the SDE4405‐ATG8c interaction (Figure S11) and do not influence the role of the SDE4405‐ATG8c interaction in activating host autophagy (Shi, Yang, et al. 2023). We then co‐expressed either WT SDE4405 or SDE4405^K87/92R^ with CsNBR1 or GUS in *N. benthamiana* leaves. CsNBR1 overexpression failed to stabilise the SDE4405^K87/92R^ mutant (Figure 4c,d). These results demonstrate that the Lys87/Lys92 site is essential for NBR1‐dependent stabilisation of SDE4405. To further investigate whether the Lys87/Lys92 sites facilitate the colonisation of *C*Las in citrus, *SDE4405*‐OE and *SDE4405* ^*K87/92R*^‐OE hairy roots were constructed respectively, with the empty vector pNMGFPer used as a control (Figure 4e and Figure S12). At 15 dpi, *C*Las titre was already detectable in *SDE4405*‐OE hairy roots. At 30 dpi, there was no significant difference in *C*Las titre between the EV group and the *SDE4405*‐OE group; however, both were significantly higher than that in the *SDE4405* ^*K87/92R*^‐OE group (Figure 4f). These results indicate that the Lys87/Lys92 sites of SDE4405 play a crucial role in promoting *C*Las colonisation.

### 2.5 CsNBR1 Disrupts Interaction of SDE4405 With CsRHY1A and Interferes With Ubiquitination of SDE4405

**FIGURE 5:**
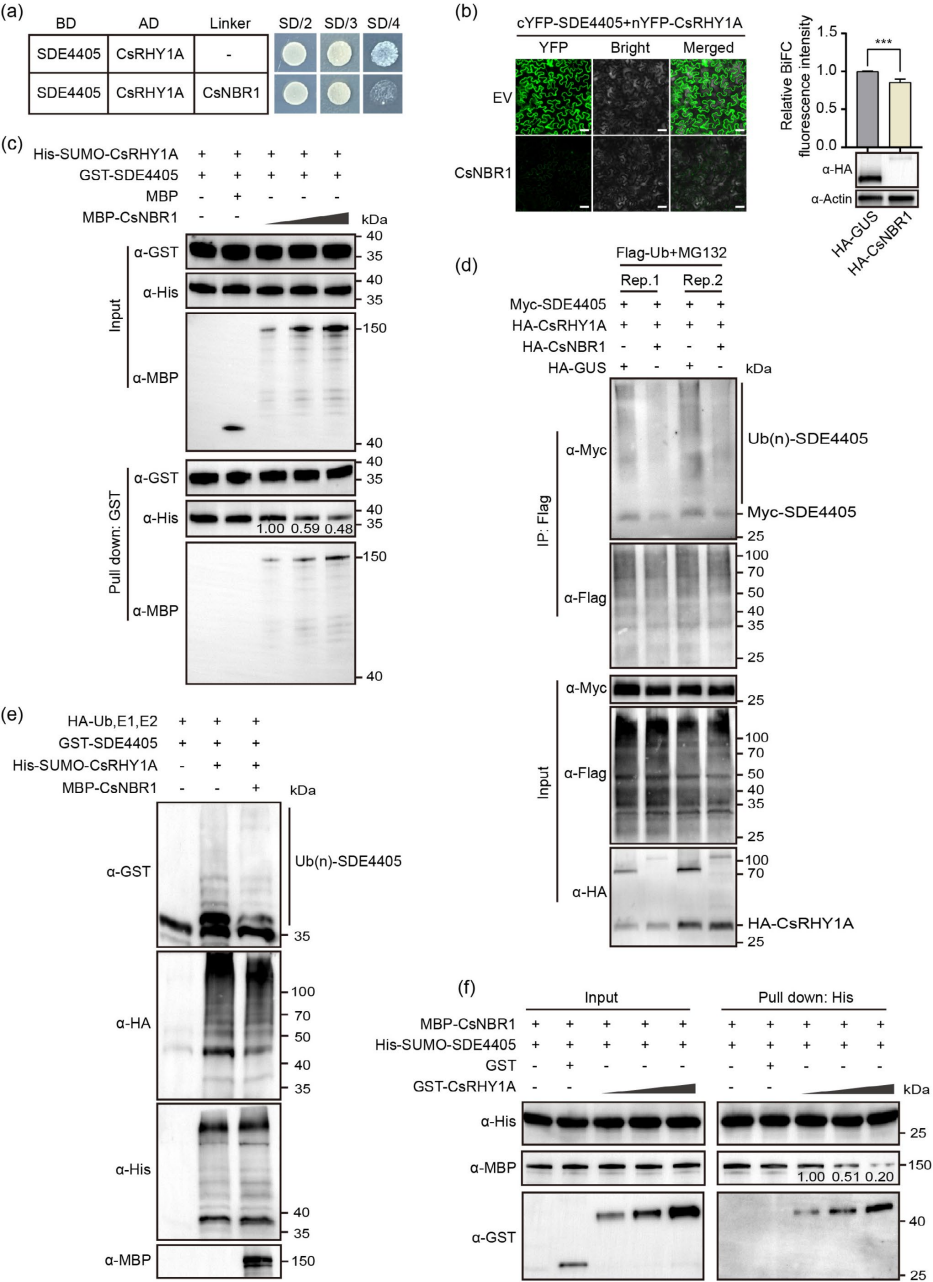
CsNBR1 disrupts SDE4405‐CsRHY1A interaction and interferes with CsRHY1A‐mediated ubiquitination of SDE4405. (a) Yeast three‐hybrid (Y3H) assay demonstrating the inhibitory effect of CsNBR1 on the SDE4405‐CsRHY1A interaction. CsNBR1 was expressed as a bridge protein. SD/2: SD/‐Leu/‐Trp. SD/3: SD/‐His/‐Leu/‐Trp. SD/4: SD/‐Met/‐His/‐Leu/‐Trp. (b) BiFC analysis showing reduced SDE4405‐CsRHY1A interaction in the presence of CsNBR1. Scale bar = 50 μm. The normalised value in expressing HA‐GUS (EV) was set to 1.00. Values are the mean ± SD of four biological replicates (***p < 0.001, Student's *t*‐test). (c) Competitive GST pull‐down assay examining the inhibitory effect of CsNBR1 on the SDE4405‐CsRHY1A interaction. Different gradient dilutions of MBP‐CsNBR1 proteins (1×, 2× and 4×) were added to the protein extractions of GST‐SDE4405 and His‐SUMO‐CsRHY1A. (d) In vivo interference analysis of CsNBR1 on the ubiquitination of SDE4405 in *Nicotiana benthamiana* leaves. Leaves were infiltrated with 25 μM MG132 at 40 hpi. Ubiquitinated SDE4405 proteins (Ubn‐SDE4405) were detected using an anti‐Myc antibody. (e) In vitro interference assays of CsNBR1 on the ubiquitination of SDE4405. Recombinant SDE4405 was incubated with E1, E2, HA‐Ub, His‐SUMO‐CsRHY1A, and MBP‐CsNBR1. Reactions were incubated at 30°C for 2 h. (f) Competitive His pull‐down assay for the inhibitory effects of CsRHY1A on the SDE4405‐CsNBR1 interaction. Different gradient dilutions of GST‐CsRHY1A proteins (1×, 2× and 4×) were added to the protein extractions of His‐SUMO‐SDE4405 and MBP‐CsNBR1. Similar results were obtained from three independent biological experiments.

Given that the interfaces of SDE4405 interact with both CsNBR1 and CsRHY1A, we determined whether the formation of the SDE4405‐CsNBR1 complex competitively modulates the capacity of CsRHY1A to degrade SDE4405. The yeast three‐hybrid (Y3H) experiment showed that the interaction intensity between SDE4405 and CsRHY1A was significantly weakened on SD/‐His/‐Leu/‐Trp/‐Met when CsNBR1 was used as a linker (Figure 5a). Consistently, the BiFC fluorescence intensity of SDE4405‐CsRHY1A was largely reduced with CsNBR1 supplementation (Figure 5b). In addition, we confirmed the competitive protein–protein interaction by in vitro pull‐down assays. GST‐SDE4405 was incubated with His‐SUMO‐CsRHY1A and increasing MBP‐CsNBR1 concentrations. The enrichment of CsRHY1A in the SDE4405‐bound resins gradually decreased with the increase in the MBP‐CsNBR1 concentration added to the system (Figure 5c). Taken together, our results suggest that CsNBR1 and CsRHY1A compete to bind with SDE4405, thus disrupting the formation of the SDE4405‐CsRHY1A subcomplex.

We showed that CsRHY1A interacted with and ubiquitinated SDE4405 (Figure 1) and that CsNBR1 interfered with the interaction between CsRHY1A and SDE4405 (Figure 5a–c). We determined whether CsNBR1 could disrupt the ubiquitination of SDE4405 by CsRHY1A in vivo and in vitro. CsNBR1 addition significantly diminished the ubiquitination of SDE4405 (Figure 5d,e). During natural HLB infection, the expression levels of both CsNBR1 and CsRHY1A are significantly upregulated; however, the fold induction of CsNBR1 is markedly higher than that of CsRHY1A (Figure S13a,b). Furthermore, the CsNBR1 protein levels were significantly increased in *C*Las‐infected plants compared to healthy plants (Figure S13c). These results reveal that *C*Las infection inhibits autophagy activity and upregulates CsNBR1, antagonising CsRHY1A‐mediated ubiquitination of SDE4405 and promoting its stability.

To investigate the mechanism by which CsRHY1A overexpression enhances *C*Las resistance, we conducted an in vitro co‐incubation assay with GST‐SDE4405, MBP‐CsNBR1, and gradient concentrations of His‐SUMO‐CsRHY1A. The interaction between CsNBR1 and SDE4405 was progressively reduced with increasing CsRHY1A concentrations (Figure 5f). These findings demonstrate that disease resistance in transgenic *CsRHY1A* plants results from the interference with SDE4405‐mediated hijacking of CsNBR1.

### 2.6 SDE4405 Suppresses CsNBR1‐Mediated Autophagic Degradation of *C*Las Effector Protein

**FIGURE 6:**
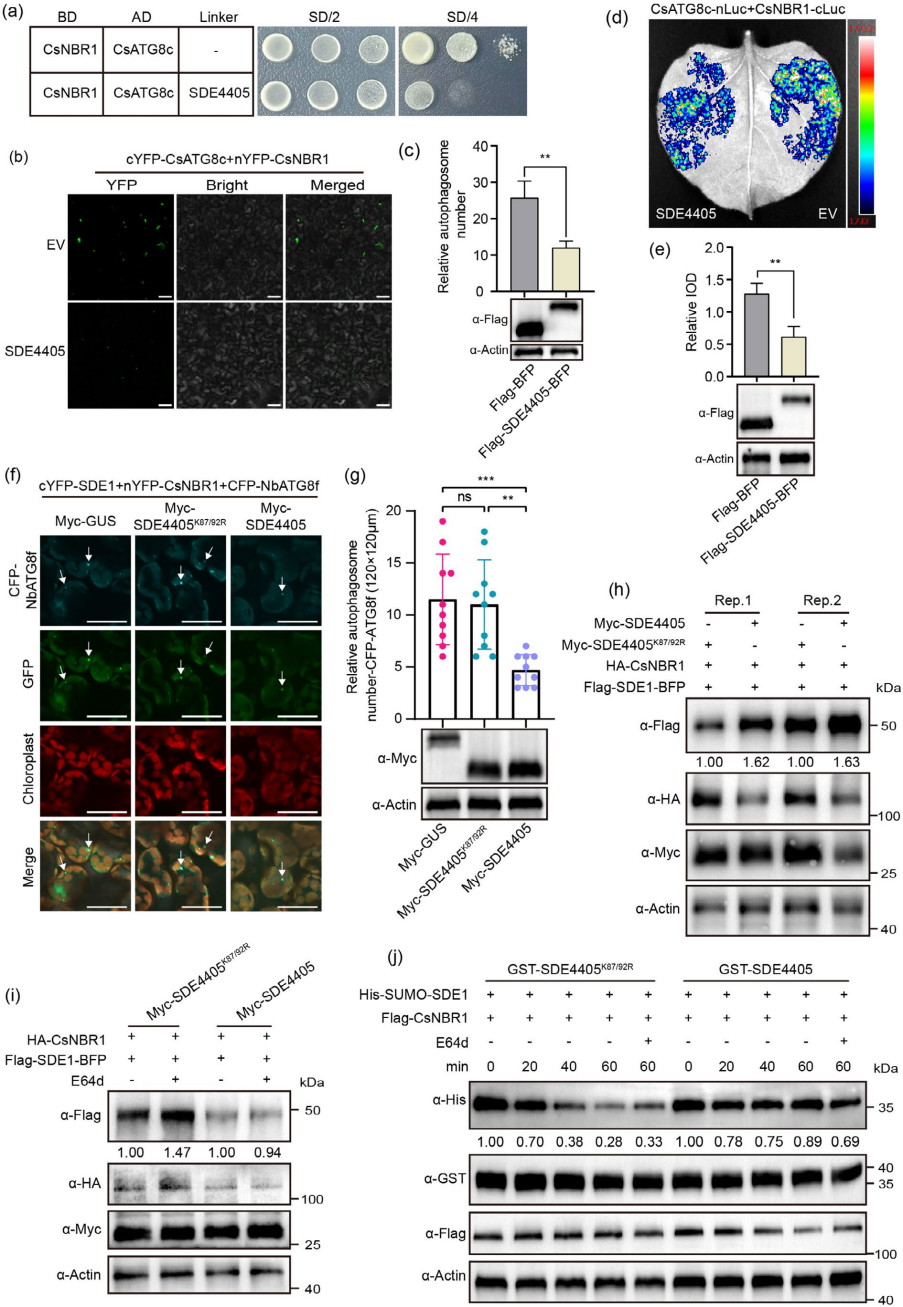
SDE4405 inhibits the degradation of SDE1 by CsNBR1‐mediated selective autophagy. (a) Y3H assay demonstrating the inhibitory effect of SDE4405 on the CsNBR1‐CsATG8c interaction. SDE4405 was expressed as a bridge protein. (b, c) Competitive BiFC assays demonstrating SDE4405 inhibition of CsNBR1‐CsATG8c‐mediated autophagosome formation. Scale bar = 50 μm. The normalised value in expressing Flag‐BFP (EV) was set to 1.00. Values are the mean ± SD of four biological replicates (**p < 0.01, Student's *t*‐test). (d, e) Luciferase complementation imaging (LCI) assay showing SDE4405 inhibition of CsNBR1‐CsATG8c interaction. The normalised value in expressing Flag‐BFP (EV) was set to 1.00. Values are the mean ± SD of four biological replicates (**p < 0.01, Student's *t*‐test). (f, g) Confocal microscopy analysis of autophagosome co‐localization with nYFP‐CsNBR1 and cYFP‐SDE1 in the presence or absence of SDE4405. cYFP‐SDE1 was co‐expressed with nYFP‐CsNBR1 and CFP‐NbATG8f (autophagosome marker) in *Nicotiana benthamiana* leaves. Myc‐GUS, Myc‐SDE4405^K87/92R^ or Myc‐SDE4405 was co‐expressed as a linker. Scale bar = 50 μm. Arrows indicate autophagosomes. The number of autophagosomes in Myc‐GUS‐expressing leaves was set to 1.0. Values are the mean ± SD of 10 biological replicates (**p < 0.01, ***p < 0.001, one‐way ANOVA). (h–j) Impact of SDE4405 on CsNBR1‐mediated autophagic degradation of SDE1. SDE1 was co‐expressed with CsNBR1 and SDE4405 in *N. benthamiana* leaves (h, i). 100 μM E64d was infiltrated into the leaves at 36 hpi (i). Total protein extracts from Flag‐*CsNBR1*‐OE citrus were incubated with His‐SUMO‐SDE1 recombinant proteins for 0, 20, 40, and 60 min with or without GST‐SDE4405. E64d was used to stop the degradation in samples collected at the pointed time (j). Similar results were obtained from three independent biological experiments.

Previous studies have shown that NBR1 functions as an autophagic receptor, binding to ubiquitinated virulence effector proteins for autophagy degradation (Leong et al. 2022; Svenning et al. 2011). However, in this study, NBR1 promoted SDE4405 protein accumulation (Figure 3e,f and Figure S9g,j). We hypothesised that SDE4405 inhibits the selective autophagy function of CsNBR1. The Y3H assays showed that the interaction intensity between CsNBR1 and CsATG8c was significantly weakened when SDE4405 was used as a linker (Figure 6a). Competitive BiFC assays revealed a reduction in the fluorescence intensity of the yellow fluorescent protein (YFP) generated by the CsNBR1‐CsATG8c interaction and a decrease in the number of autophagosomes (Figure 6b,c). Furthermore, the competitive luciferase complementation imaging (LCI) assays revealed a decrease in the fluorescence intensity of the CsNBR1–CsATG8c interaction after SDE4405 introduction (Figure 6d,e). These results collectively suggest that SDE4405 disrupts the binding of CsNBR1 and CsATG8c, thereby inhibiting CsNBR1‐mediated selective autophagy.

We then determined whether SDE4405 could interfere with CsNBR1‐mediated autophagic degradation of other *C*Las effector proteins. Using BiFC and LCI assays, we showed that SDE1 (CLIBASIA_05315), a previously identified virulence effector protein, interacted with CsNBR1 (Figure S14a,b) (Clark et al. 2018). The CFP‐ATG8f protein is a structural component of autophagosomes and is widely used to label them (Tong et al. 2023; Yang et al. 2021). cYFP‐SDE1 and nYFP‐CsNBR1 were co‐expressed with CFP‐NbATG8f in *N. benthamiana* leaves with or without SDE4405. Few YFP granules were reconstituted by cYFP‐SDE1 and nYFP‐CsNBR1 in SDE4405‐treated samples, likely due to reduced autophagosome formation (Figure 6f,g). Transient co‐expression of SDE1 with CsNBR1 in *N. benthamiana* decreased the protein abundance of SDE1 compared to the control (Figure S14c). E64d treatment significantly increased SDE1 protein accumulation, whereas MG132 treatment failed to alter their stability (Figure S14d). These results indicate that SDE1 was degraded by CsNBR1‐mediated selective autophagy, whereas SDE4405 prevented SDE1 degradation by binding to NBR1 (Figure 6h,i). Cell‐free degradation assays revealed that SDE4405 effectively suppressed CsNBR1‐mediated autophagic degradation of SDE1, with WT SDE4405 maintaining significantly extended substrate half‐lives compared to the ubiquitination‐deficient SDE4405^K87/92R^ mutant (Figure 6j). The transcript abundance of SDE1 was quantified in WT and *CsRHY1A*‐OE *C*Las infected‐plants and showed that the transcript levels of SDE1 were significantly reduced in transgenic lines compared to WT controls (Figure S14e). Taken together, these findings demonstrate that SDE4405 suppresses autophagic degradation of virulence factor SDE1 by subverting CsNBR1‐mediated selective autophagy machinery, which facilitates *C*Las pathogenesis.

## 3 Materials and Methods

### 3.1 Plant Materials and Chemical Treatments

*Nicotiana benthamiana* plants were cultivated at 25°C (day/night) in an insect‐free greenhouse under long‐day conditions (16 h light/8 h dark). Transgenic and WT ‘Wanjincheng’ ( *C. sinensis* ) plants were maintained in a greenhouse set at 28°C. *SDE4405*‐OE plants were generated as previously described (Shi, Yang, et al. 2023), were planted in the National Citrus Germplasm Repository, Chongqing, China.

For the protein abundance assay, the uppermost one or two fully expanded leaves from 4‐week‐old *N. benthamiana* plants were inoculated with 100 μM CHX (MedChemExpress), 50 μM MG132 (MedChemExpress), 100 μM E64d (MedChemExpress), and the corresponding control, dimethyl sulfoxide (DMSO) (Hu et al. 2023).

### 3.2 Plasmid Construction

To obtain the coding sequence (CDS) of CsNBR1 and CsRHY1A, cDNA from *C. sinensis* was used as a template for amplification. The CDS of SDE4405 was amplified according to the previous description (Shi, Yang, et al. 2023). All CDS were introduced into binary expression vectors, controlled by CaMV 35S promoter and NOS terminator, with or without protein tags. Plasmids were created through homologous recombination for transient expression in *N. benthamiana*, resulting in fusion vectors labelled with GFP, BFP, nYFP and cYFP.

For stable citrus transformation, PCR‐amplified CDS without stop codons of CsNBR1 and CsRHY1A were inserted into the CaMV 35S‐driven pLGNe or pNmGFPer vector. To generate RNAi constructs, a 200–300 bp fragment of CDS in target genes was amplified and cloned into pNmGFPer vector for gene silencing. The confirmed vectors were transformed into citrus plants by *Agrobacterium*‐mediated citrus epicotyl transformation according to a previously described protocol (Du et al. 2022).

For VIGS assays, the TRV‐CsNBR1 plasmid was constructed by cloning a 317 bp sequence targeting the *CsNBR1* gene into the TRV2 vector.

For Y2H analysis, the mentioned genes were amplified and subsequently inserted into pGADT7 (AD) and pGBKT7 (BD) vectors, which were utilised to generate constructs for Y2H experiments. To ensure the accuracy of these constructs, sequencing was performed to verify the integrity of the vectors.

For *Escherichia coli* expression vectors, the protein‐coding regions of SDE4405, CsNBR1 and CsRHY1A were individually cloned into pGEX6P, pET28a or pMAL‐C5X vectors. Then, the vectors were transformed into *E. coli* Rosetta (DE3) strain for recombinant protein expression. The primers used for vector plasmid construction were listed in Supporting Information: Table S2.

### 3.3 *Agrobacterium*‐Mediated Transient Expression Assays

*Agrobacterium*‐mediated transient expression was performed in *N. benthamiana*. Four‐week‐old *N. benthamiana* plants were infiltrated with *A. tumefaciens* strain EHA105 harbouring target constructs. Following overnight culture, bacterial cells were centrifuged at 5000 rpm for 5 min and resuspended in MMA buffer (10 mM MES, 10 mM MgCl_2_, pH 5.6, and 200 μM acetosyringone) to an appropriate OD_600_. Leaf samples were collected 48 hpi, and transient protein expression was detected by immunoblotting with specific antibodies as previously described (Wang, Li, et al. 2023).

### 3.4 *C. sinensis* Hairy Root Transformation

The constructs of pNmGFPer‐*CsNBR1*‐OE, pNmGFPer‐*CsRHY1A*‐OE, or pNmGFPer‐*CsRHY1A*‐RNAi were introduced into *SDE4405*‐OE or Citrange plants via *A. rhizogenes* K599‐mediated transformation as previously described (Ma et al. 2022; Ramasamy et al. 2023). Gene transformation identification and *C*Las graft‐inoculation were performed at approximately 120 dpi.

### 3.5 Protein–Protein Interaction Assays

For the Y2H assay, the transformed cells were plated on synthetic defined (SD) medium (SD/‐Leu‐Trp) (Coolaber, PM2220) and incubated for 3 days at 30°C. Subsequently, they were cultured on selection medium (SD/‐His‐Leu‐Trp) (Coolaber, PM2150) to assess the interaction.

For the BiFC assay, *A. tumefaciens* GV3101 carrying pCV‐nYFP and pCV‐cYFP derivatives were mixed in a 1:1 ratio at a final OD_600_ value of 0.5 and then transformed into *N. benthamiana* leaves. Fluorescence was observed at 2 dpi using a laser scanning confocal microscope (Olympus‐FV3000, Japan). BiFC fluorescent signals were quantified using methods previously described (Kudla and Bock 2016).

For the LCI assay, *A. tumefaciens* GV3101 carrying the nLuc and cLuc constructs was mixed in a 1:1 ratio and adjusted to an OD_600_ of 0.5 in the induction medium. The agroinfiltrated *N. benthamiana* leaves were treated with 150 μg/mL d‐luciferin (Yeasen, Shanghai, China) at 48 h post‐infiltration and incubated for 5 min and imaged as previously described (Zhou et al. 2018).

For the Co‐IP assay, Myc‐SDE4405 was co‐expressed with HA‐CsNBR1 or HA‐CsRHY1A and HA‐GUS in *N. benthamiana* leaves via *A. tumefaciens* ‐mediated transient expression. After 48 hpi, total proteins were extracted with Plant Protein Extraction Kit (Solarbio, bc3720, Beijing Solarbio Science & Technology) and precipitated with anti‐GFP beads (Proteintech) following the product manual. Immunoblotting was then carried out to detect both the total proteins (input) and the precipitated proteins (output) using anti‐HA (Cat No. 003‐101‐005, Alpvhhs) or anti‐Flag antibodies (Cat No. 016‐303‐005, Alpvhhs).

For the GST‐pull down assay, the His‐, MBP‐, GST‐fused recombinant proteins were expressed in *E. coli* Rosetta (DE3) strain and purified with GST‐tag Protein Purification Kit (Beyotime) or His‐tag Protein Purification Kit (Beyotime), following the manufacturer's instructions. GST‐SDE4405 was used to pull down interacting proteins as previously described (Hu et al. 2023). The pulled‐down proteins were detected by immunoblotting with anti‐MBP or anti‐His antibodies (Proteintech).

### 3.6 In Vitro and In Vivo Ubiquitination Assays

In vitro ubiquitination assay of CsRHY1A was performed as described with slight modification (Sun et al. 2024; Wang et al. 2024). 200 ng of GST‐CsRHY1A or GST‐CsRHY1A^H173/176Y^ recombinant protein was mixed in the presence or absence of 0.25 μg of E1 (Cat No. E‐305, R&D Systems), 0.5 μg of E2 (Cat No. E2‐627, R&D Systems), and 1.25 μg of HA‐Ub (Cat No. U‐110, R&D Systems) in a ubiquitination buffer (80 mM Tris–HCl [pH 7.5], 25 mM MgCl_2_,12 mM ATP; 30 μL per sample) and incubated, at 30°C for 2 h. Proteins were separated on a 10% (w/v) gradient SDS‐PAGE gel (EpiZyme) under nonreducing conditions. Immunodetection was carried out using anti‐GST (Proteintech) or anti‐HA antibodies, respectively. For in vitro CsRHY1A‐mediated SDE4405 ubiquitination assay, GST‐CsRHY1A and MBP‐SDE4405 proteins or MBP protein control were co‐incubated as described previously (Hu et al. 2023). The reaction products were analysed using anti‐GST and anti‐MBP antibodies.

For in vivo ubiquitination assay, Myc‐SDE4405, Myc‐SDE4405^K87R^, Myc‐SDE4405^K92R^ and Myc‐SDE4405^K87/92R^, together with HA‐CsRHY1A and Flag‐Ub were co‐expressed in 4‐week‐old *N. benthamiana* leaves with 25 μM MG132. All the ubiquitinated proteins were immunoprecipitated using an anti‐Flag antibody, the ubiquitination of Myc‐SDE4405, Myc‐SDE4405^K87R^, Myc‐SDE4405^K92R^, and Myc‐SDE4405^K87/92R^ was detected by immunoblotting the immunoprecipitated products with an anti‐Myc antibody (Cat No. 002‐203‐005, Alpvhhs).

To evaluate the effect of CsNBR1 on the ubiquitination of SDE4405 mediated by CsRHY1A in vitro, MBP‐CsNBR1 was added to the ubiquitination reaction mixture containing GST‐SDE4405, E1, E2, HA‐Ub and His‐SUMO‐CsRHY1A. The mixture was incubated at 30°C for 2 h, and the reaction products were analysed by immunoblotting using anti‐GST, anti‐HA, anti‐His, or anti‐MBP antibodies.

To investigate the effect of CsNBR1 on CsRHY1A‐mediated ubiquitination of SDE4405 in vivo, Myc‐SDE4405 and HA‐CsRHY1A together with HA‐CsNBR1 (or HA‐GUS as a negative control) were co‐infiltrated with Flag‐Ub into *N. benthamiana* leaves. Total protein extracts were immunoprecipitated using anti‐Flag magnetic beads, and ubiquitinated Myc‐SDE4405 was detected by immunoblotting with an anti‐Myc antibody.

### 3.7 Mass Spectrometry Determination of Ubiquitination Sites

SDE4405‐GFP were expressed in *N. benthamiana* leaves for 48 h and immunoprecipitated as previously described (Wang et al. 2024). Bead‐bound proteins were released by boiling, separated via SDS‐PAGE, and target bands were excised, trypsin‐digested, and subjected to LC–MS/MS analysis. The MS/MS spectra were analysed with Q‐Exactive plus (ThermoFisher Scientific).

### 3.8 In Vivo and Cell‐Free Degradation Assays

In vivo degradation assays were conducted with minor modifications as described (Liu et al. 2023). *Agrobacterium* EHA105, harbouring either Flag‐ or Myc‐tagged SDE4405 plasmids, and *Agrobacterium* EHA105, carrying HA‐CsRHY1A or HA‐CsNBR1 plasmids, were co‐transfected into *N. benthamiana* or citrus plants. The empty vector pART27, which expresses an HA‐GUS protein, was co‐transfected as an internal control. Leaves from *N. benthamiana* or citrus were collected at 2 dpi, and 50 μM MG132 was injected into the corresponding leaves 12 h prior to sampling.

Cell‐free degradation assays were performed as previously described (Wang et al. 2024, 2022). Protein extracts were prepared from the leaves of 4‐week‐old *N. benthamiana* plants transiently expressing HA‐CsRHY1A or HA‐CsRHY1A^H173/176Y^ recombinant proteins, as well as from *CsRHY1A*‐OE or RNAi citrus plants, using the Plant Protein Extraction Kit (Solarbio, bc3720, Beijing Solarbio Science & Technology). The purified recombinant His‐SUMO‐SDE4405 (200 ng) and His‐SUMO‐SDE4405^K87/92R^ (200 ng) proteins were then incubated with protein extracts at 28°C, with or without 50 μM MG132. Reactions were terminated via addition of 1× protein loading buffer and incubating at 95°C for 10 min. Immunoblot analysis was performed using an anti‐His antibody, with Actin and initial recombinant His‐SUMO‐SDE4405 (input) serving as loading controls.

### 3.9 Confocal Observation of Autophagosomes, Autophagic Bodies

The related constructs and the autophagy marker CFP‐ATG8f were co‐infiltrated into *N. benthamiana* leaves. The samples were observed and imaged using a confocal microscope (Olympus‐FV3000, Japan) at 48 hpi. The number of autophagic bodies and autophagosomes was counted by ImageJ (Leong et al. 2022).

### 3.10 RNA Extraction and Gene Expression Analysis

Total RNA was extracted with an RNAiso Plus (TaKaRa) and reverse‐transcribed to cDNA using an All‐In‐One 5× RT MasterMix (ABM). RT‐qPCR assays were performed using BlasTaq 2X qPCR MasterMix (ABM) on a q225 real‐time PCR machine (Novogene). The actin gene was used as an internal reference to calculate the relative expression of target genes. The *C*Las‐carrying scion segments are grafted onto the rootstock close to the scion, and samples are taken near the virus source for bacterial titers detection. The bacterial titers (*C*Las cells per μg of citrus DNA) were quantified with a qPCR assay that was described by Zou et al. (2017): *C*Las titre = 10^(−0.2718×CtLas16S+10.624)^/10^(−0.2749×CtCs18S+4.0531)^ × 1000. Gene‐specific primers are listed in Supporting Information: Table S2.

### 3.11 Quantification and Statistical Analysis

Protein band signals were quantified by using ImageJ software. All experiments were performed with at least three biological replicates. Statistical significance analysis was examined by Student's *t*‐test, one‐way ANOVA, or two‐way ANOVA using GraphPad software (Wang et al. 2024). Relative gene expression was calculated using the 2^−ΔΔCt^ method (Livak and Schmittgen 2001).

## 4 Discussion

In this study, we explored the *C*Las–host interaction using effector SDE4405 as a probe, which revealed dual functionality of SDE4405 in modulating autophagy and ubiquitination pathways. We found that the SDE4405 protein is tightly regulated by the 26S proteasome degradation pathway and that the RING domain of the E3 ligase CsRHY1A is required to regulate SDE4405 homeostasis. However, SDE4405 interacted with CsNBR1 and competed with CsRHY1A at the SDE4405 ubiquitination sites. Upon *C*Las infection, the transcriptional and translational upregulation of CsNBR1 promoted its competitive binding to SDE4405 at Lys87/92 residues, thereby conferring SDE4405 stability.

Ubiquitination and autophagy pathways serve as critical battlegrounds in the arms race between plants and pathogens. Many E3 ligase genes involved in plant defence responses have been reported. For example, OsRFPH2‐10 promotes rice dwarf virus (RDV) P2 degradation and is involved in antiviral defence during the early infection stages (Liu et al. 2014). OsRGLG5 is targeted by *Magnaporthe oryzae* effector AvrPi9 and positively regulates basal resistance against rice blast (Liu et al. 2023). We recently demonstrated that the overexpression of a dominant‐negative form of E3 ligase PUB21, named PUB21DN, stabilises MYC2 and confers HLB resistance in citrus (Zhao et al. 2025). In this study, we identified E3 ligase CsRHY1A, which contains a C3‐H2‐C3 type RING‐finger domain located near the C‐terminal of the protein. RING‐finger motifs are characterised by conserved Cys and His residues that form cross‐brace‐arranged free loops and bind two Zn cations (Borden 2000; Shen et al. 2016). SDE4405 degradation was not mediated in CsRHY1A^H173/176Y^, the E3 ligase activity‐defective mutant, demonstrating that the RING finger is required for the E3 ligase activity of CsRHY1A (Figure 1g,h). Considering the competitive interactions among CsRHY1A, CsNBR1, and SDE4405 and the upregulated expression of CsNBR1 during *C*Las infection, we generated transgenic *CsRHY1A*‐overexpressing citrus lines, establishing CsRHY1A as a positive regulator of plant defence against *C*Las.

NBR1, one of the best‐studied selective autophagy receptors, is an important target regulated by pathogen effectors (Kirkin et al. 2009; Svenning et al. 2011). *Xcv* effector XopL, which has E3 ligase activity, can ubiquitinate and degrade the autophagy component SH3P2, disrupting autophagy. However, XopL is targeted by NBR1‐triggered selective autophagy, enhancing plant defence (Leong et al. 2022). The geminivirus satellite‐encoded protein βC1 interacts with NbNBR1 to form cytoplasmic granules, thereby preventing βC1 degradation triggered by E3 ligase NbRFP1 (Zhou et al. 2021). We identified two ubiquitination sites, Lys87 and Lys92, for the SDE4405 effector, and they are required for its interaction with both CsRHY1A and CsNBR1 and essential for both CsRHY1A‐mediated SDE4405 ubiquitination and NBR1‐dependent SDE4405 stabilisation (Figure 4a–d and Figure S4). Furthermore, these two sites are essential for SDE4405 to promote *C*Las colonisation in citrus (Figure 4f). Consistent with a previous study (Zhou et al. 2021), the SDE4405‐CsNBR1 interaction also resulted in cytoplasmic granules (Figure S7b). CsNBR1 competitively bound to Lys87/92 of SDE4405, thereby blocking ubiquitination by E3 ligase CsRHY1A and evading CsRHY1A‐mediated proteasomal degradation. These findings reveal a novel regulatory strategy evolved in bacterial effectors.

NBR1 interacts with ATG8 protein to initiate selective autophagic degradation of target proteins (Guo et al. 2023; Hofius et al. 2017; Zhang and Chen 2020). In mammalian cells, NIMA‐related kinase 9 (NEK9)‐mediated phosphorylation of the microtubule‐associated LC3B protein at Thr‐50 suppresses selective autophagy of p62 (Shrestha et al. 2020). In *P. infestans* , RXLR effector PexRD54 antagonises NBR1/Joka2 for binding to ATG8CL and interferes with the Joka2‐ATG8CL complex, inhibiting selective autophagic degradation (Dagdas et al. 2016). It is unclear whether interference with the NBR1‐ATG8 complex disrupts the recruitment of other virulence effectors for autophagic degradation. We identified a *C*Las effector, SDE1 (CLIBASIA_05315), that interacts with CsNBR1 and is degraded by CsNBR1‐mediated selective autophagy (Figure S14). SDE4405 suppressed the interaction between NBR1 and ATG8c, disrupting NBR1‐dependent autophagic degradation of virulence effector SDE1 (Figure 6). Our analysis provides a seminal example, showing that a bacterial effector can hijack a selective cargo receptor for its own stabilisation and protects other effectors of the same pathogen from autophagic degradation, thus facilitating pathogen infection. Further investigations should determine whether SDE4405‐mediated disruption of NBR1‐ATG8 binding impairs the autophagic degradation of additional pathogen effectors targeted by NBR1 to confer broad‐spectrum immune evasion capabilities.

**FIGURE 7:**
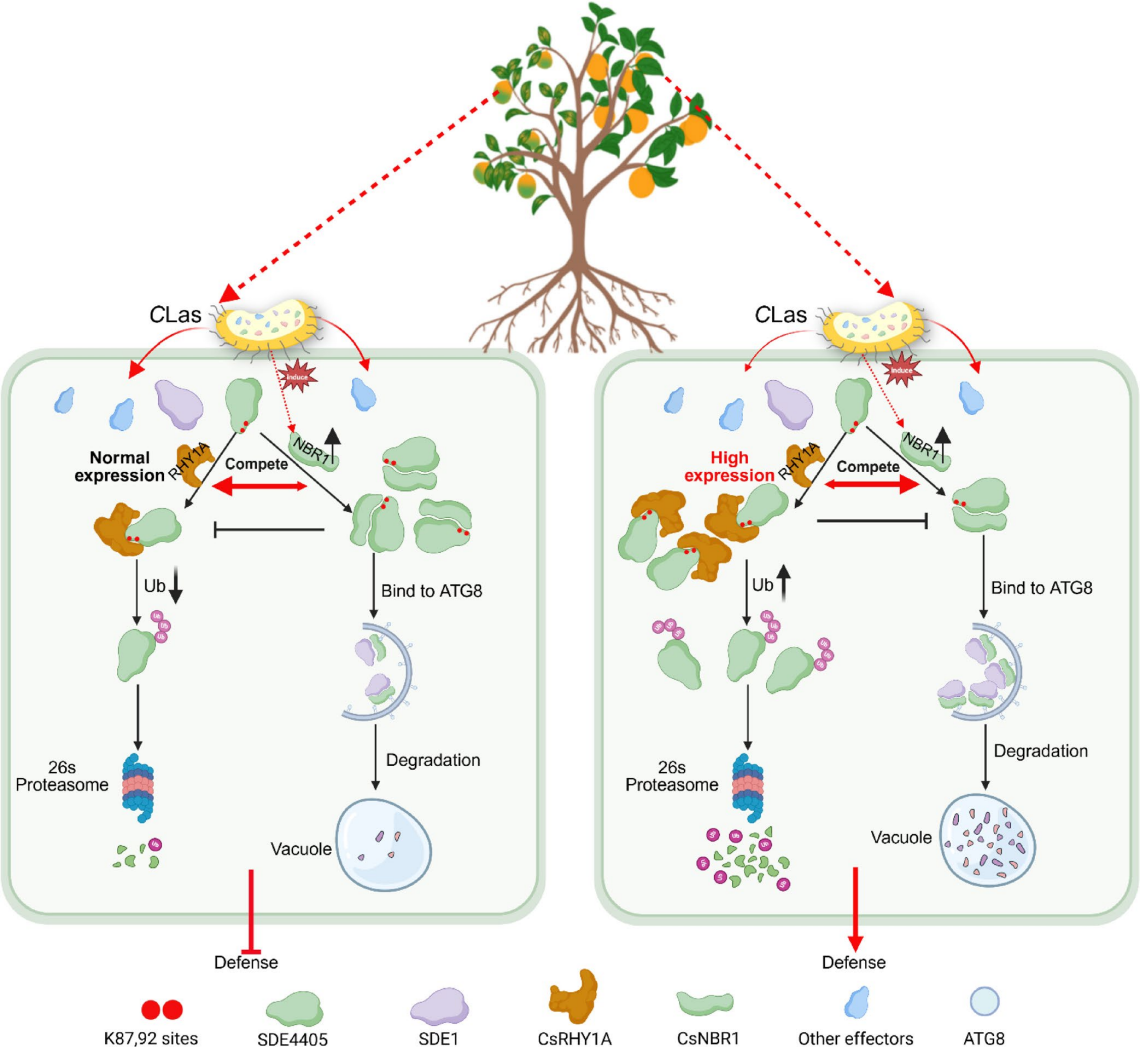
The working model of SDE4405 hijacking CsNBR1 to evade autophagic and CsRHY1A‐mediated proteasomal degradation. Upon *C*Las infection, the transcription and translation of CsNBR1 are upregulated. In WT citrus (left), the *C*Las‐secreted effector SDE4405 manipulates the host protein CsNBR1 to reduce its ubiquitination, escaping 26S proteasome‐dependent degradation, thereby maintaining its protein stability. SDE4405 also disrupts the binding of CsNBR1 and CsATG8c, thereby inhibiting CsNBR1‐mediated autophagic degradation of effector protein SDE1, which suppresses basal defence and facilitates pathogen infection. In *CsRHY1A* overexpression citrus (right), CsRHY1A competitively binds to SDE4405 at Lys87/92 residues, promoting SDE4405 ubiquitination and proteasomal degradation. SDE1 is degraded by CsNBR1‐mediated selective autophagy, ultimately enhancing plant defence against *C*Las infection.

In summary, we propose a working model illustrating how interactions among CsNBR1, CsRHY1A, and SDE4405 dynamically regulate *C*Las pathogenicity in planta (Figure 7). During infection, *C*Las‐secreted effector SDE4405 is recognised by E3 ligase CsRHY1A, leading to its ubiquitination and subsequent degradation, which attenuates bacterial virulence. To counteract host defences, SDE4405 competitively binds to autophagy receptor CsNBR1 at Lys87/92 residues, impairing CsRHY1A‐mediated ubiquitination and stabilising the effector protein. SDE4405 also suppresses CsNBR1‐mediated selective autophagic degradation of *C*Las effector protein. These approaches reveal a novel bacterial strategy for subverting host immunity, which has great potential for the further design of disease‐resistance breeding programmes.

## Author Contributions

X.W. conceived and supervised the study. Y.S. performed cell‐free protein degradation assay, produced the constructs and generated transgenic plants. Y.S., F.F., X.L. and H.S. performed Y2H assays Co‐IP, Pull‐down, BiFC, LCI, and subcellular localization. Y.S., F.F. and Z.Y. performed RNA analysis and western blotting. Z.Y. and X.C. purified the recombinant proteins. X.W. and Y.S. wrote the paper. C.Z. supervised the paper. All authors have read and approved the paper submission.

## Funding

This work was supported by grants from National Natural Sciences Foundation of China (U23A20196), National Key Research and Development Programme of China (2021YFD1400800), Special Fund for Youth Team of Southwest University (SWU‐XJLJ202310), Innovation Research 2035 Pilot Plan of Southwest University (SWU‐XDZD22002) and Southwest University research and innovation project (SWUB24080).

## Conflicts of Interest

Patent relating to this work and their usage have been filed by Southwest University (China patent, 202510823806.9). The authors declare no conflicts of interest.

## Supporting information


**Figure S1:** SDE4405 is subjected to 26S proteasomal degradation.
**Figure S2:** CsRHY1A and NbRHY1A degrades SDE4405 via the 26S proteasome system.
**Figure S3:** Bioinformatic analysis of CsRHY1A.
**Figure S4:** SDE4405^K87/K92^ was critical for CsRHY1A‐mediated ubiquitination.
**Figure S5:** Positive identification of *CsRHY1A* transgenic hairy roots.
**Figure S6:** Positive identification of *CsRHY1A* transgenic citrus plants.
**Figure S7:** Localization analysis of SDE4405 and CsNBR1.
**Figure S8:** SDE4405 interacts with the UBA domain of CsNBR1.
**Figure S9:** NbNBR1 interacts with and stabilises SDE4405.
**Figure S10:** Molecular identification of *CsNBR1* overexpression (*CsNBR1*‐OE) citrus hairy roots.
**Figure S11:** Lys87 and Lys92 are not the interaction sites between SDE4405 and ATG8c.
**Figure S12:** Identification of SDE4405 and SDE4405^K87/92R^ transgenic hairy roots by semi‐quantitative PCR.
**Figure S13:** Expression and protein accumulation profiles of CsNBR1 or CsRHY1A in *C*Las‐infected citrus.
**Figure S14:** CsNBR1‐mediated selective autophagy targets SDE1 for degradation.

**Table S1:** Candidate proteins of *Citrus sinensis* interact with SDE4405 via Y2H assays.

**Table S2:** List of primers used in this study.

## Data Availability

The data that support the findings of this study are available in Supporting Information.
