# Supplementary material for: A Bacterial Effector Hijacks NBR1 to Modulate Both Autophagy and Ubiquitination‐Mediated Degradation That Promotes Bacterial Infection
Source: Plant Biotechnol J. 2025 Dec 17;24(4):2492–509. doi: 10.1111/pbi.70509 (PMC13140610; doi:10.1111/pbi.70509)
Supplement: Supplementary file 1 — Figure S1: SDE4405 is subjected to 26S proteasomal degradation. Figure S2: CsRHY1A and NbRHY1A degrades SDE4405 via the 26S proteasome system. Figure S3: Bioinformatic analysis of CsRHY1A. Figure S4: SDE4405K87/K92 was critical for CsRHY1A‐mediated ubiquitination. Figure S5: Positive identification of CsRHY1A transgenic hairy roots. Figure S6: Positive identification of CsRHY1A transgenic citrus plants. Figure S7: Localization analysis of SDE4405 and CsNBR1. Figure S8: SDE4405 interacts with the UBA domain of CsNBR1. Figure S9: NbNBR1 interacts with and stabilises SDE4405. Figure S10: Molecular identification of CsNBR1 overexpression (CsNBR1‐OE) citrus hairy roots. Figure S11: Lys87 and Lys92 are not the interaction sites between SDE4405 and ATG8c. Figure S12: Identification of SDE4405 and SDE4405K87/92R transgenic hairy roots by semi‐quantitative PCR. Figure S13: Expression and protein accumulation profiles of CsNBR1 or CsRHY1A in CLas‐infected citrus. Figure S14: CsNBR1‐mediated selective autophagy targets SDE1 for degradation. [file PBI-24-2492-s002.docx]

**Figure S1**

**
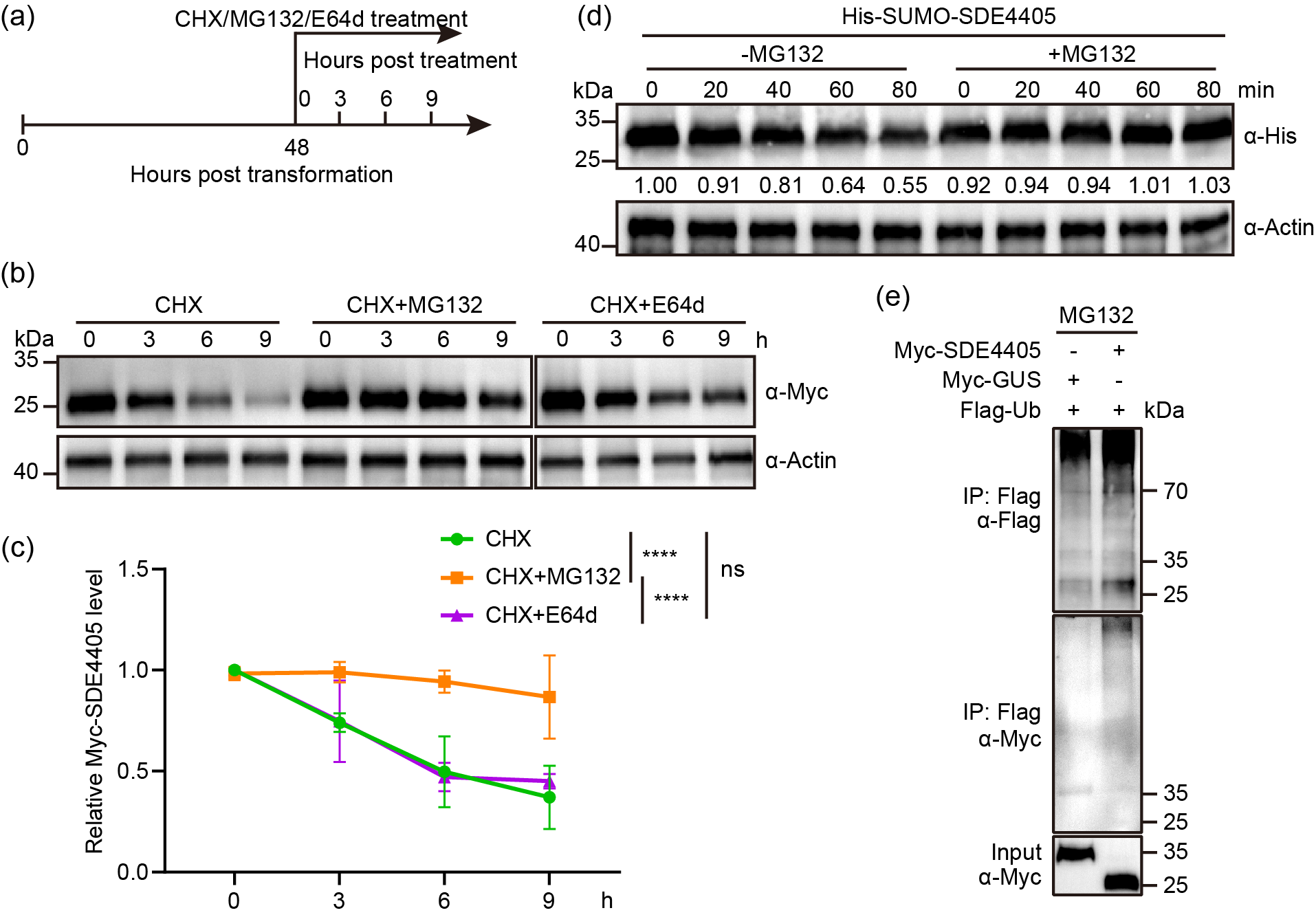
**

Figure S1. SDE4405 is subjected to 26S proteasomal degradation. **(a)** Schematic diagram illustrating the protocol for the SDE4405 *in vivo* degradation assay. **(b, c)** The impact of the 26S proteasome inhibitor MG132 or autophagy inhibitors E64d on SDE4405 levels in *Nicotiana benthamiana* leaves. *N. benthamiana* leaves transiently expressing Myc-SDE4405 were treated with 50 μM MG132, or 100 μM E64d, and/or 200 μM cycloheximide (CHX) for the specified durations. Relative Myc-SDE4405 protein abundance was quantified in ImageJ, with Actin serving as a control. Data represent mean ± SD of three biological replicates (*****P* < 0.0001, two-way ANOVA). **(d)** A cell-free degradation assay for SDE4405. Recombinant His-SUMO-SDE4405 was incubated with crude extracts from citrus leaves. Numbers under the bands indicate relative His-SUMO-SDE4405 abundance, normalized to Actin antibody membrane as a loading control. **(e)** Ubiquitination level of SDE4405 in plants. Total proteins from *N. benthamiana* leaves expressing Myc-SDE4405 or Myc-GUS with Flag-Ub were extracted, followed by immunoprecipitation (IP) with anti-Flag beads. Myc-GUS served as a negative control.

**Figure S2**

**
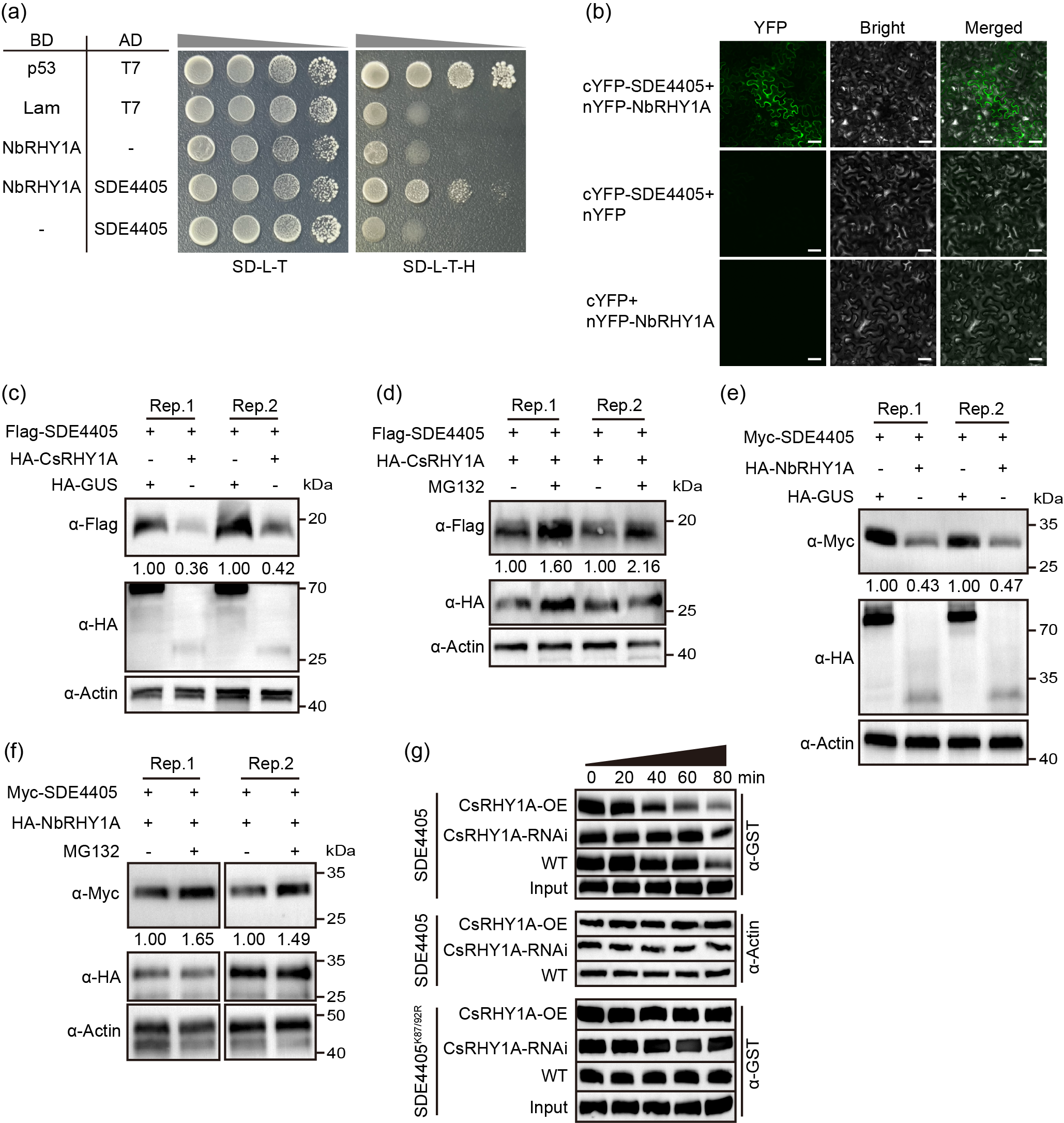
**

Figure S2. CsRHY1A and NbRHY1A degrades SDE4405 via the 26S proteasome system. **(a)** Y2H assays confirming interactions between NbRHY1A and SDE4405. **(b)** BiFC assays examining interactions between NbRHY1A and SDE4405. Scale bar = 50 μm. **(c–f)** Impact of RHY1A on SDE4405 degradation. CsRHY1A (c, d) or NbRHY1A (e, f) was co-expressed with SDE4405 in *N. benthamiana* leaves. 50 μM MG132 was infiltrated into the leaves at 36 hpi (d, f). Samples were collected for immunoblotting at 48 hpi. **(g)** *In vitro* degradation assay of SDE4405 by CsRHY1A in transgenic citrus plants. Total protein extracts from WT, *CsRHY1A*-OE, and -RNAi citrus leaves were incubated with recombinant GST-SDE4405. The reaction mixtures were analyzed by SDS-PAGE and immunoblotting with an anti-GST antibody. Input GST-SDE4405 and Actin served as loading controls.

**Figure S3**


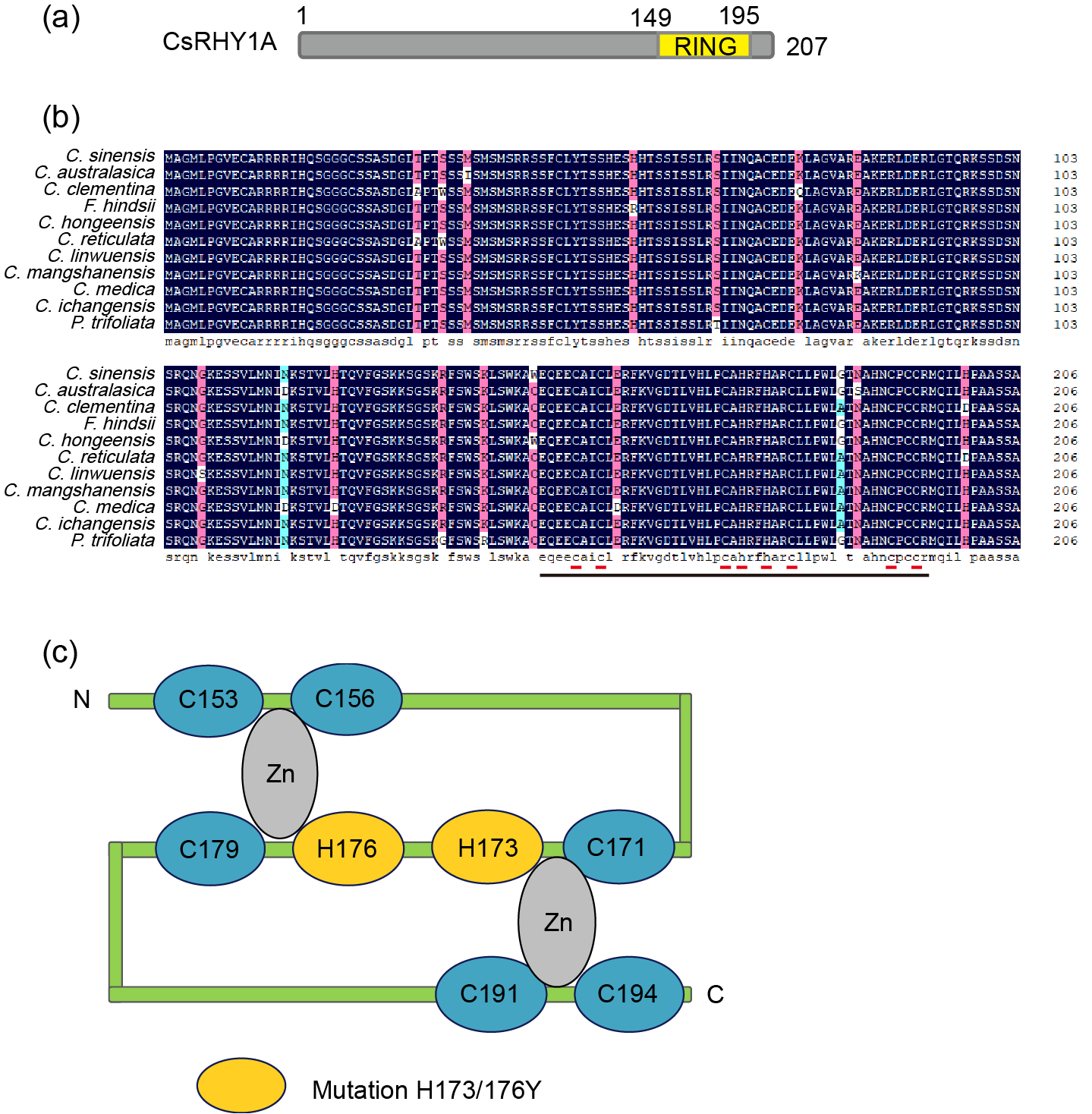


Figure S3. Bioinformatic analysis of CsRHY1A. **(a)** Amino acid structure schematic of CsRHY1A. **(b)** Multiple sequence alignment of RHY1A amino acid sequence from *Citrus* species. The black line indicates the RING domain, while the red line indicates the C3H2C3-type RING-H2 finger consensus motif (Zn^2+^ binding site). **(c)** Schematic illustration of the CsRHY1A C3H2C3 RING finger architecture and the mutated sites within the RING domain.

**Figure S4**


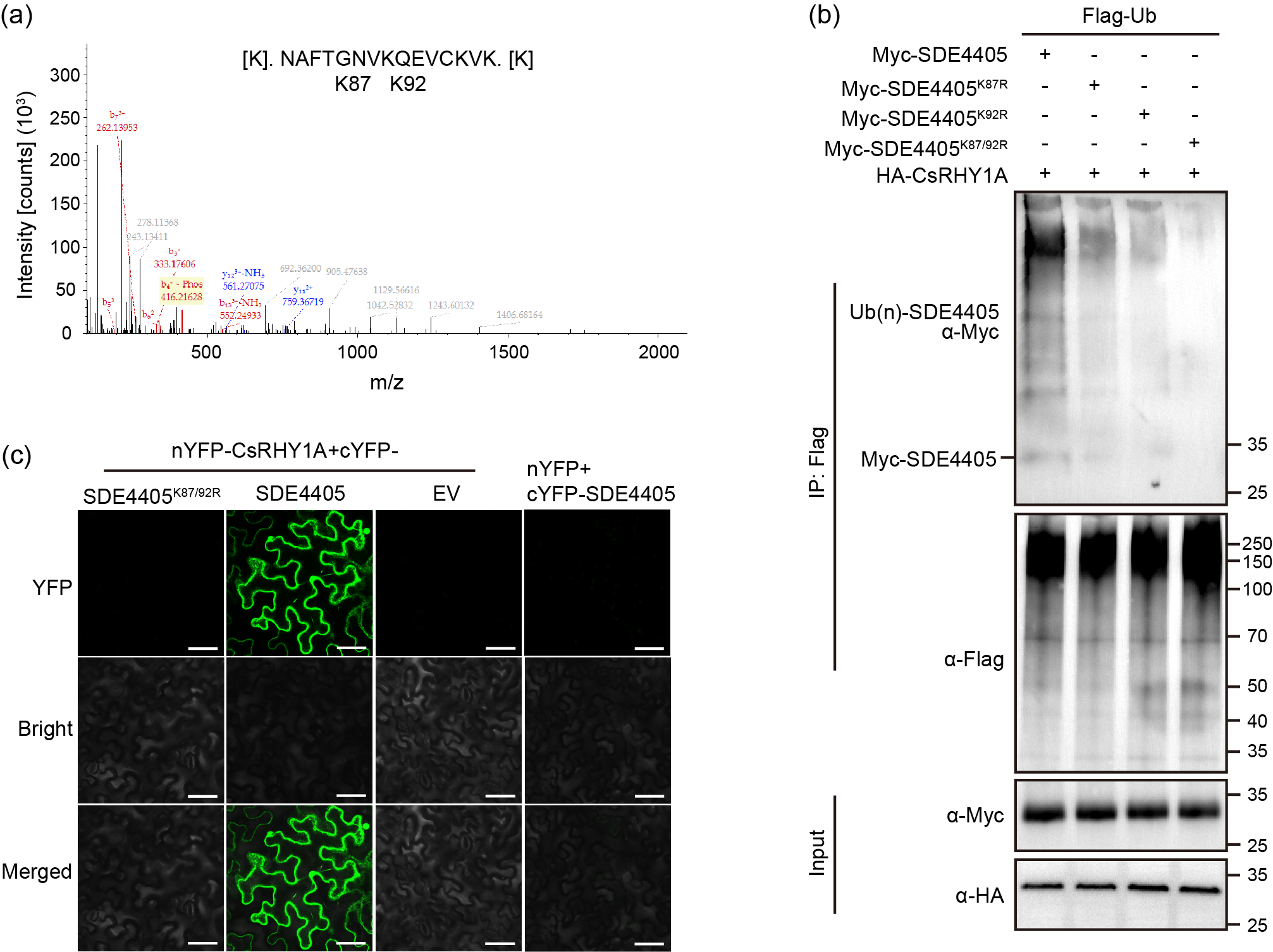
Figure S4. SDE4405^K87/K92^ was critical for CsRHY1A-mediated ubiquitination. (**a**) Identification of SDE4405 ubiquitination sites by liquid chromatography–tandem mass spectrometry (LC-MS/MS) analysis. (**b**) Reduced ubiquitination of SDE4405^K87/92R^ by CsRHY1A. HA-CsRHY1A and Flag-Ub were co-expressed with Myc-SDE4405, Myc-SDE4405^K87R^, Myc-SDE4405^K92R^, or Myc-SDE4405^K87/92R^ in *N. benthamiana*. At 40 hpi, leaves were infiltrated with 25 μM MG132 and harvested 8 h later. Ubiquitinated SDE4405 was immunoprecipitated with anti-Flag beads and detected by anti-Myc antibody. Input HA-tagged CsRHY1A proteins were confirmed by an anti-HA antibody. (**c**) BiFC analysis of CsRHY1A interaction with SDE4405^K87/92R^ in *N. benthamiana* leaves. nYFP-CsRHY1A and cYFP-SDE4405^K87/92R^ were co-infiltrated, and confocal imaging was performed at 48 hpi. Scale bar = 50 μm.

**Figure S5**


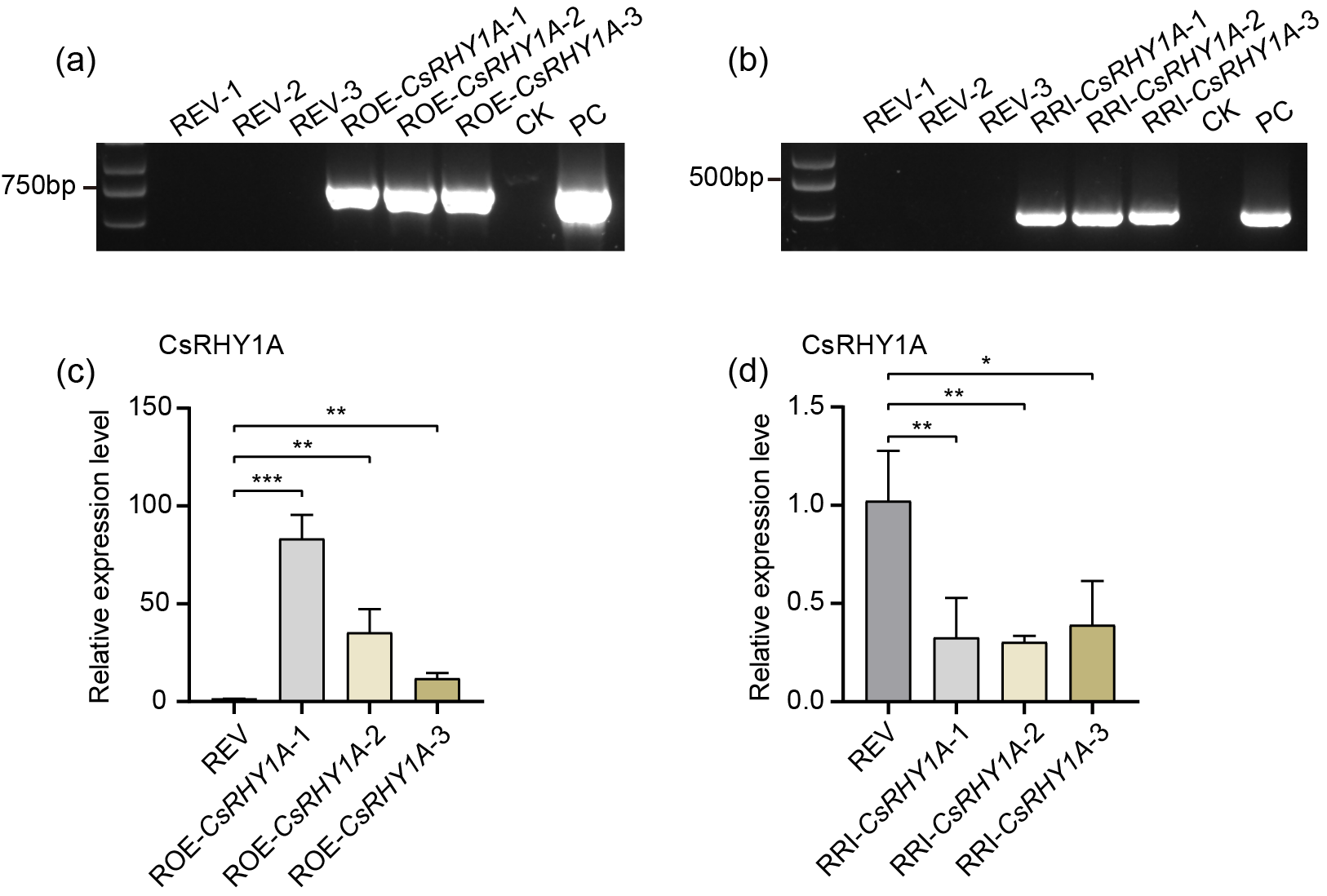


Figure S5. Positive identification of *CsRHY1A* transgenic hairy roots. (a, b) Semi-quantitative PCR analysis of CsRHY1A expression in ROE-*CsRHY1A* (a) and RRI-*CsRHY1A* (b) hairy root lines. REV (negative control); Sterile double-distilled water (CK, control check); The target gene plasmid (PC, positive control). (c, d) Relative mRNA levels of CsRHY1A in ROE-*CsRHY1A* (c) and RRI-*CsRHY1A* (d) citrus hairy roots. Values are means ± SD (n = 3) (**P* < 0.05, ***P* < 0.01, Student’s *t*-test).

**Figure S6**

**
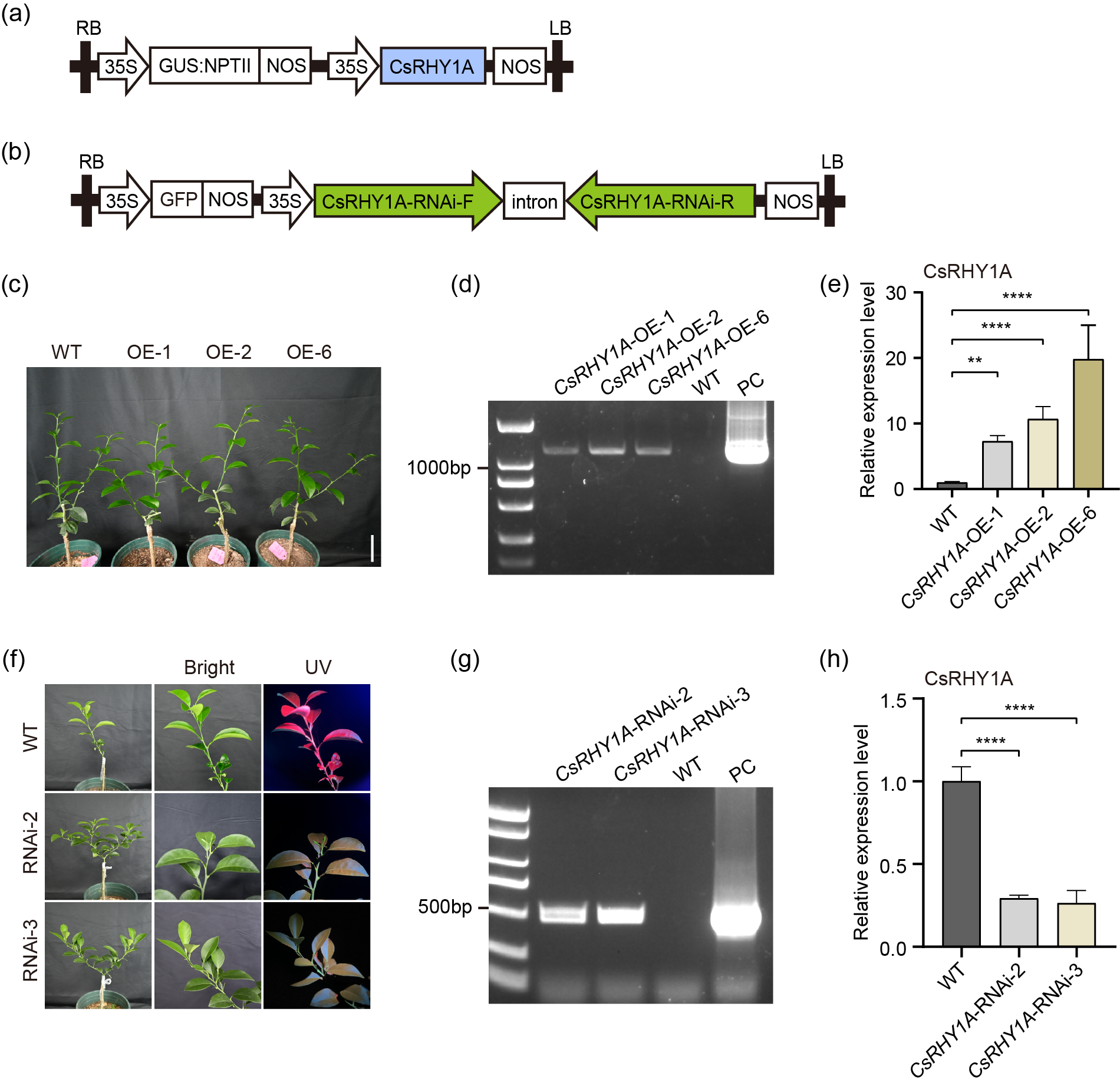
**

Figure S6. Positive identification of *CsRHY1A* transgenic citrus plants. **(a, b)** Diagram of the construction of *CsRHY1A*-OE (a) or -RNAi (b) recombinant expression vector. **(c)** Phenotypic analyses of *CsRHY1A*-OE plants. **(d)** Analysis of the expression of CsRHY1A in overexpressed citrus lines by semi-quantitative PCR. The target gene plasmid (PC, positive control). Scale bar = 10 cm. **(e)** Relative mRNA levels of CsRHY1A was evaluated in overexpressed plants. Values are means ± SD (n = 3) (***P* <0.01, *****P* <0.0001, one-way ANOVA). **(f)** Phenotypes of *CsRHY1A*-RNAi citrus lines. Under blue light, transgenic plants displayed green fluorescence, whereas WT citrus emitted red fluorescence. **(g)** Analysis of the expression of CsRHY1A in RNAi citrus lines by semi-quantitative PCR. PC, positive control. **(h)** Relative mRNA levels of CsRHY1A were evaluated in RNA interfered plants. In e and h, *CsActin* was used as a control gene for normalization. Values are means ± SD (n = 3) (*****P* <0.0001, one-way ANOVA).

**Figure S7**


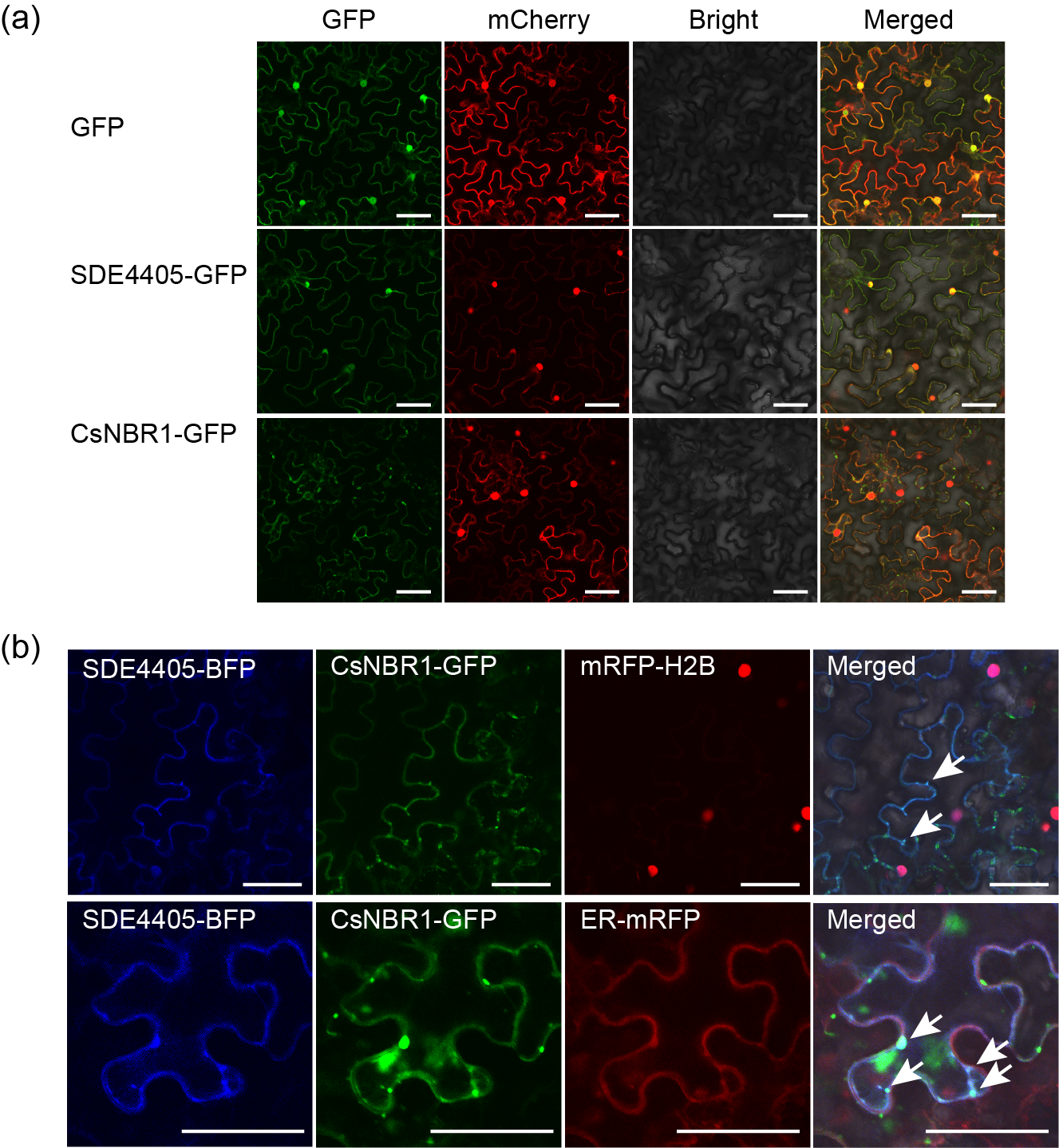


Figure S7. Localization analysis of SDE4405 and CsNBR1. **(a)** Subcellular localization of SDE4405 and CsNBR1 in *N. benthamiana* epidermis cells. mCherry was used as a marker of nuclear and plasma membrane localization. Scale bar = 50 μm. **(b)** Co-localization of SDE4405 and CsNBR1 in *N. benthamiana* epidermis cells. H2B: a marker of nuclear localization; ER: a marker of endoplamic reticulum localization. Scale bar = 50 μm. All experiments were repeated three times with similar results.

**Figure S8**


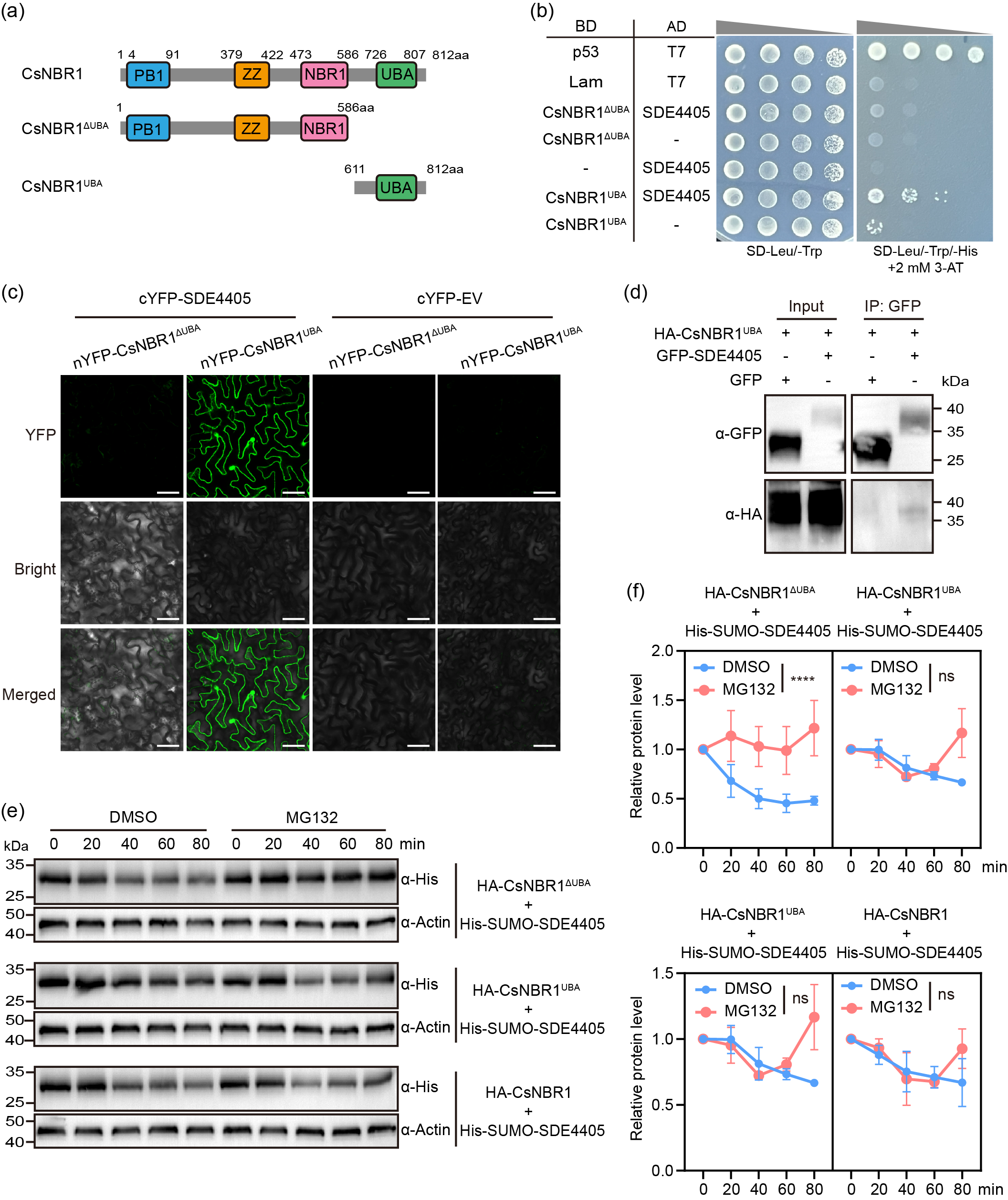


Figure S8. SDE4405 interacts with the UBA domain of CsNBR1. **(a)** Schematic illustration of full-length CsNBR1 and its deletion derivatives. aa: amino acid. **(b)** Y2H analysis of SDE4405 binding to truncated CsNBR1 variants. **(c)** BiFC assays demonstrating the interactions of truncated CsNBR1 with SDE4405 in *N. benthamiana*. Scale bar = 50 μm. **(d)** Co-IP analysis of CsNBR1^UBA^-SDE4405 interaction. CsNBR1^UBA^ was co-expressed with GFP-SDE4405 or GFP in *N. benthamiana* leaves. Total proteins were immunoprecipitated with anti-GFP beads and detected by an anti-HA antibody. **(e, f)** Representative Western blot images (e) and quantification of three independent experiments (f) of cell-free degradation assays. Protein extracts were prepared from *N. benthamiana* leaves expressing HA-CsNBR1, HA-CsNBR1^ΔUBA^, and HA-CsNBR1^UBA^ incubated with His-SUMO-SDE4405 and treated with DMSO or MG132 (50 μM). The abundance of SDE4405 was examined by immunoblotting with an anti-His antibody, using an anti-Actin antibody as a loading control. Values are means ± SD (n = 3) (ns indicates no significant differences; *****P* < 0.0001, two-way ANOVA). MG132 was used to stop the degradation in samples collected at the pointed time. The experiments were repeated at least three times with similar results.

Figure S9


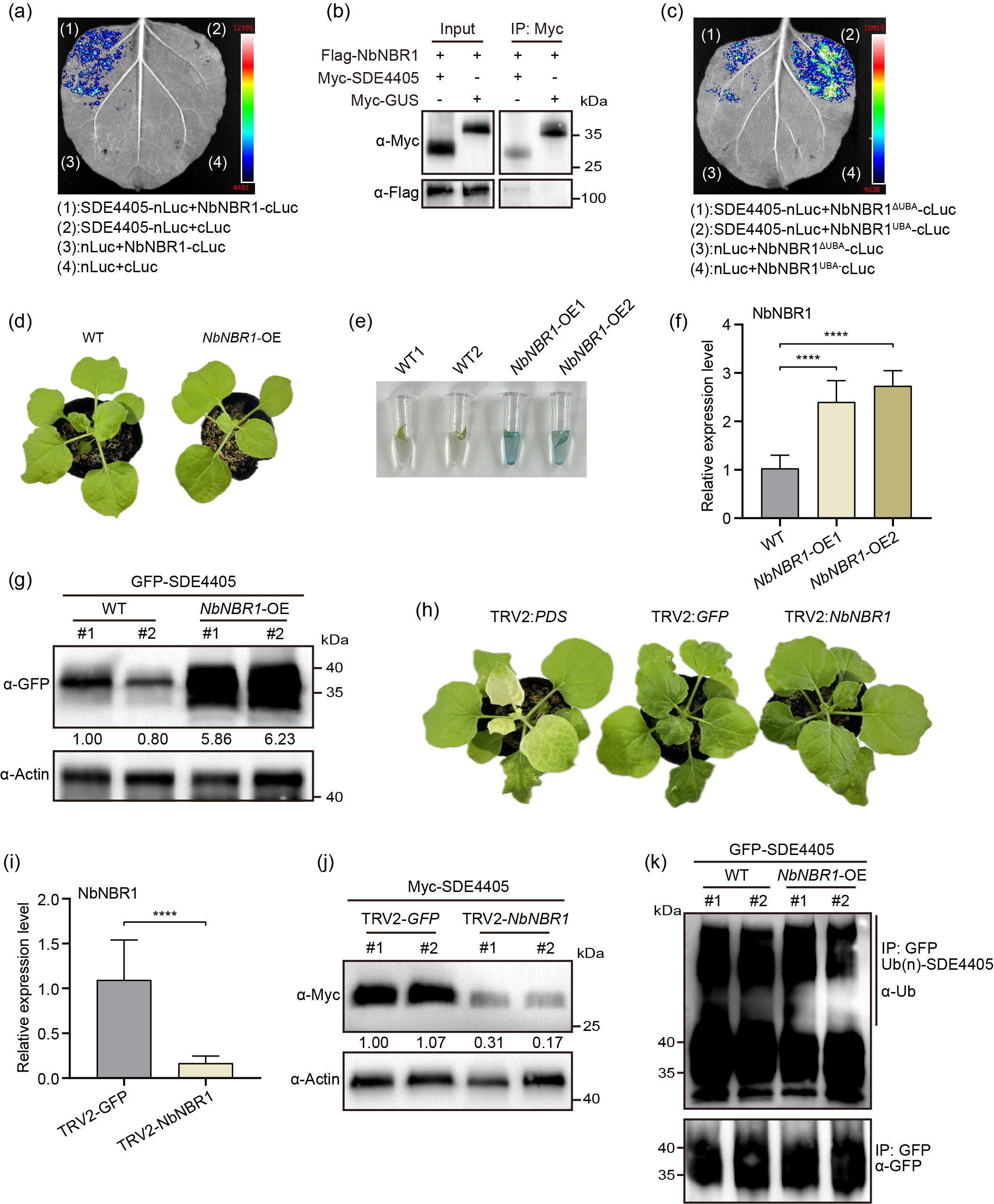


Figure S9. NbNBR1 interacts with and stabilizes SDE4405. (a) LCI assays demonstrating potential interactions between SDE4405 and NbNBR1. (b) Co-IP assays of the SDE4405-NbNBR1 interaction. Total proteins were extracted and incubated with anti-Myc beads. Input and immunoprecipitated proteins were detected by immunoblotting with anti-Myc and anti-Flag antibodies. (c) LCI assay demonstrating interactions between SDE4405 and the UBA domain of NbNBR1. (d) Phenotypes of WT and *NbNBR1* overepression (*NbNBR1*-OE) *N. benthamiana* plants. (e) GUS staining confirmation of *NbNBR1*-OE plant lines. (f) Relative NbNBR1 mRNA levels in *NbNBR1*-OE *N. benthamiana* leaves, quantified by real-time quantitative polymerase chain reaction (RT-qPCR) and normalized to *NbActin*. Values are means ± SD (n = 3) (*****P* < 0.0001, one-way ANOVA). (g) Immunoblot analysis of SDE4405-GFP protein levels in stable transgenic *N. benthamiana* expressing 35S:SDE4405-GFP in *NbNBR1*-OE and WT backgrounds. Protein accumulation was detected with an anti-GFP antibody, and Actin served as a loading control. (h) Phenotypes of *N. benthamiana* plants infected with TRV2-*PDS* (positive control), TRV2-*GFP* (negative control), or TRV2-*NbNBR1* (silencing construct). (i) RT-qPCR analysis of NbNBR1 silencing efficiency in TRV2-*NbNBR1* plants. Values are means ± SD (n = 3) (*****P* < 0.0001, Student’s *t*-test). (j) Immunoblot analysis of Myc-SDE4405 protein levels in TRV2-*NbNBR1* or TRV2-*GFP* *N. benthamiana* leaves, detected using an anti-Myc antibody. (k) Ubiquitination levels of SDE4405 expressed in *NbNBR1*-OE and WT *N. benthamiana* leaves. SDE4405-GFP was immunoprecipitated from total protein extracts using anti-GFP magnetic beads, and ubiquitination was detected by immunoblotting with an anti-Ub antibody.

**Figure S10**

**
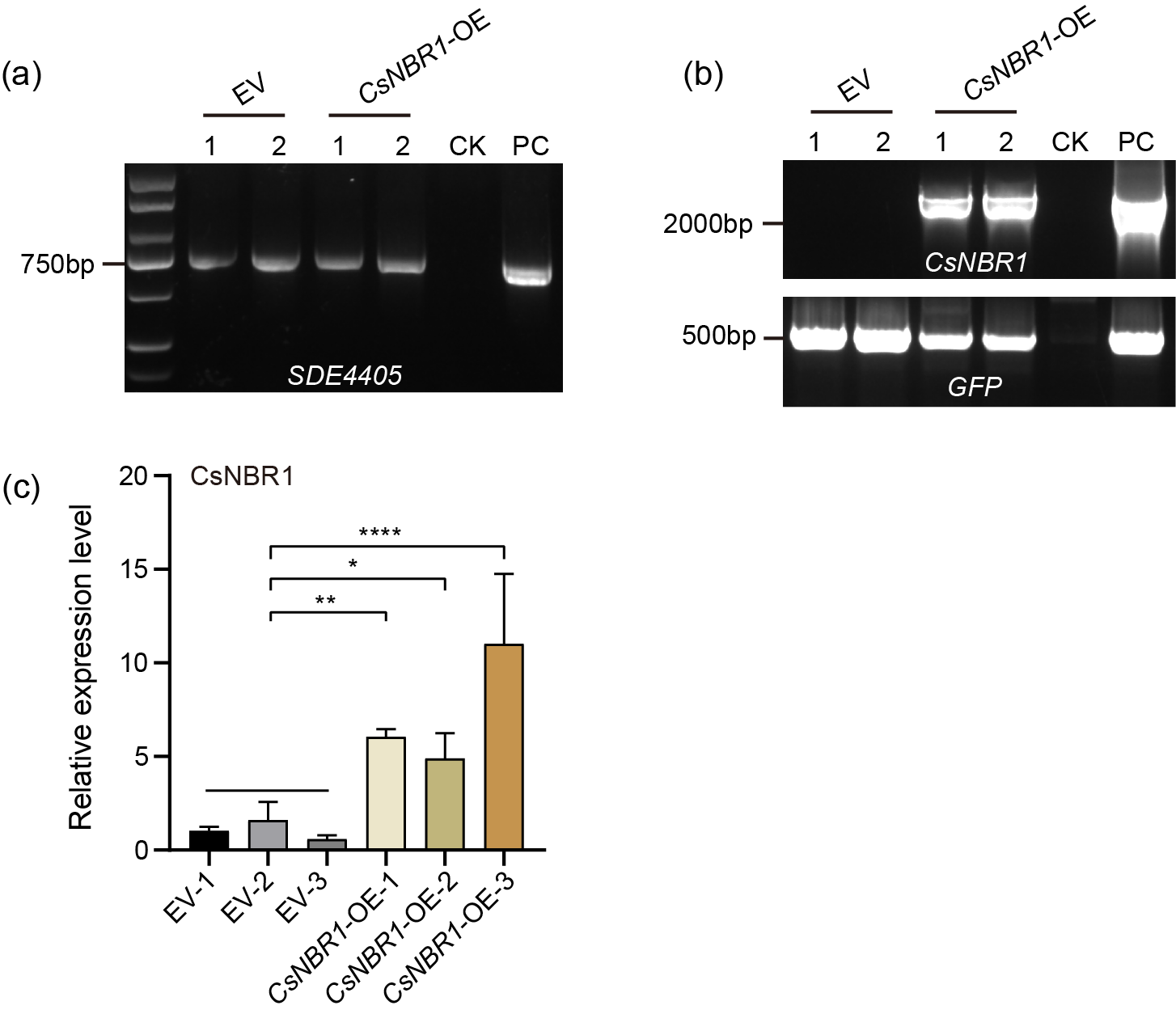
**

Figure S10. Molecular identification of *CsNBR1* overexpression (*CsNBR1*-OE) citrus hairy roots. (a) Semi-quantitative PCR diagnostic image of SDE4405 in transgenic hairy roots produced by *C*Las-free *SDE4405*-OE citrus. (b) Semi-quantitative PCR detection of CsNBR1 transgene and GFP reporter gene in transgenic hairy roots. (c) Relative mRNA levels of CsNBR1 in *CsNBR1*-OE citrus hairy roots. Values are means ± SD (n = 3) (**P* < 0.05, ***P* < 0.01, *****P* < 0.0001, one-way ANOVA).

**Figure S11**


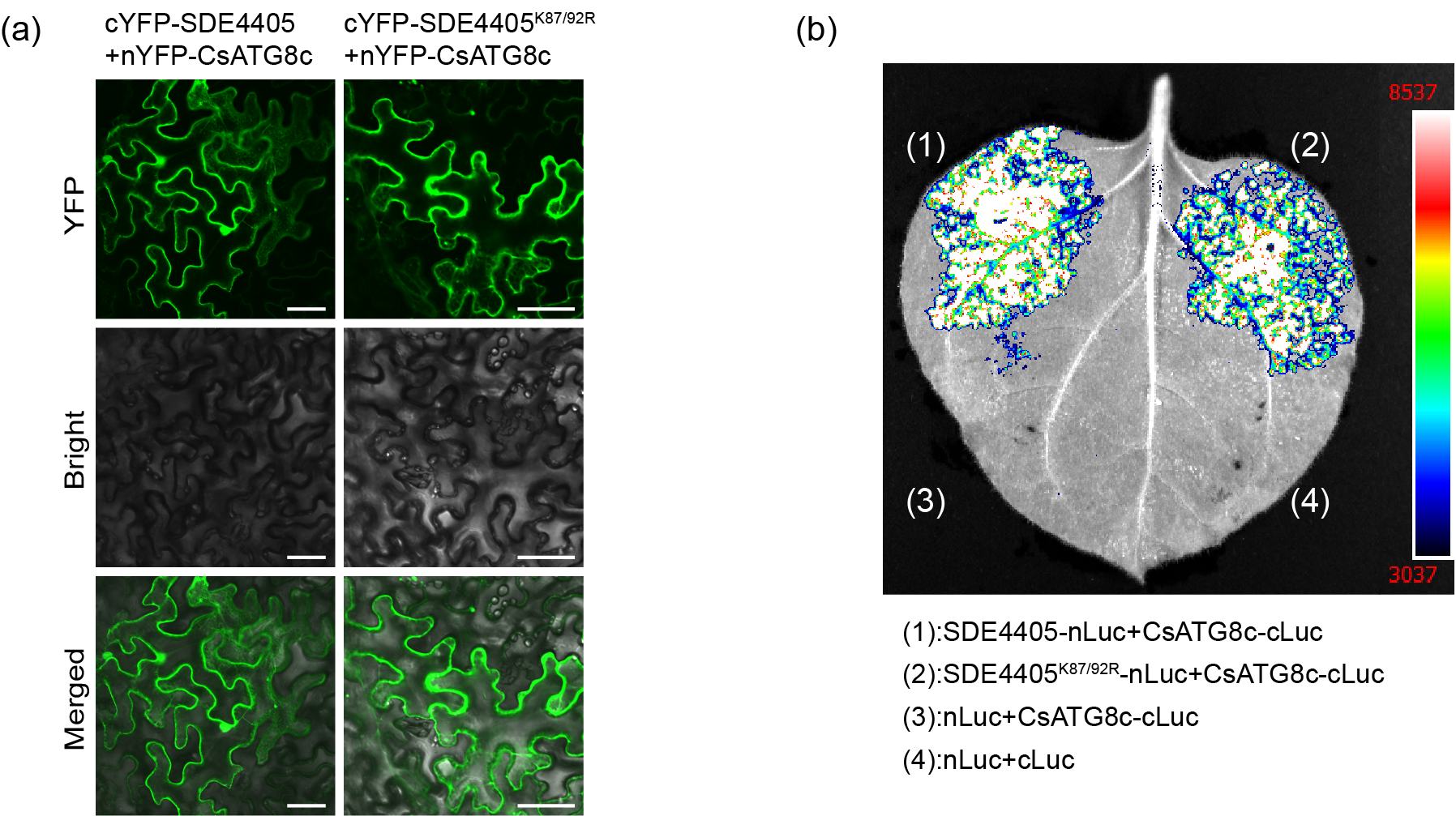


Figure S11. Lys87 and Lys92 are not the interaction sites between SDE4405 and ATG8c. (a) BiFC assays examining interactions between SDE4405^K87/92R^ and CsATG8c. *Agrobacterium* carrying the indicated recombinant constructs was inoculated to four-week-old fully expanded *N. benthamiana* leaves. YFP fluorescence was visualized using confocal microscopy at 48 hpi. Scale bar = 50 μm. (b) LCI assays of SDE4405^K87/92R^-CsATG8c interaction in *N. benthamiana* leaves. SDE4405 and SDE4405^K87/92R^ were fused to the N-terminus of nLUC to generate SDE4405/SDE4405^K87/92R^-nLUC; CsATG8c was fused to the C-terminus of cLUC to generate CsATG8c-cLUC.

**Figure S12**

**
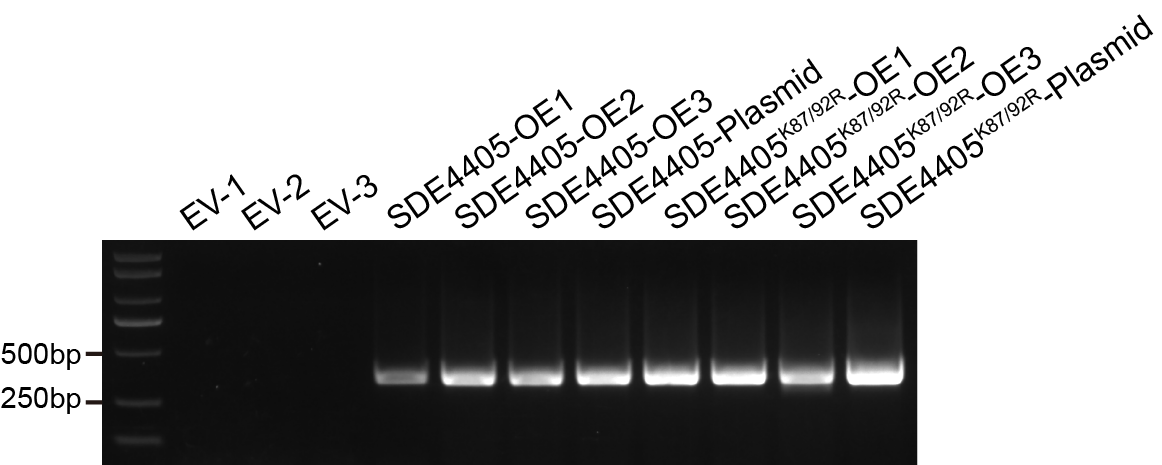
**

**Figure S12.** Identification of SDE4405 and SDE4405^K87/92R^ transgenic hairy roots by semi-quantitative PCR.

**Figure S13**

**
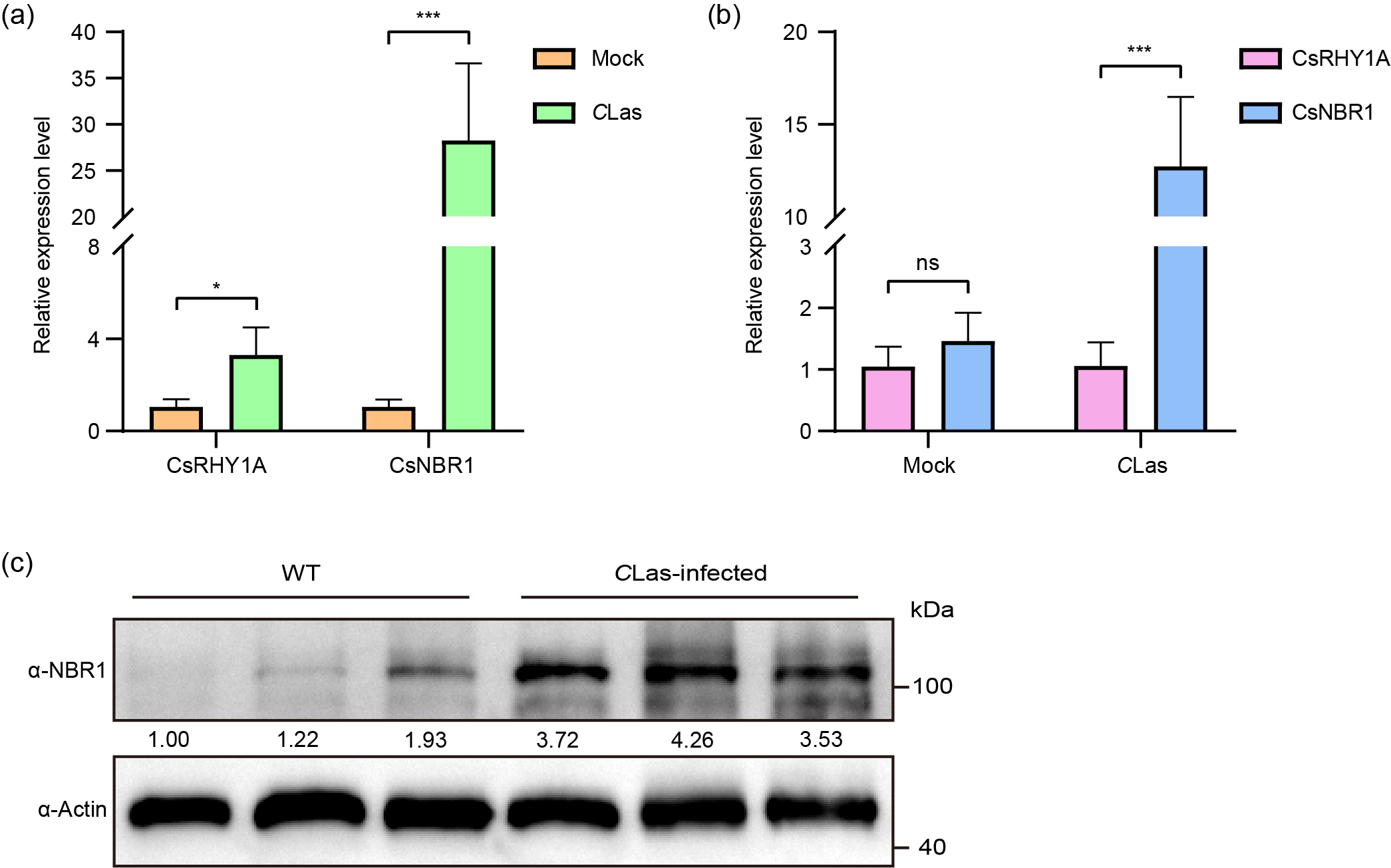
**

Figure S13. Expression and protein accumulation profiles of CsNBR1 or CsRHY1A in *C*Las-infected citrus. (a, b) Relative mRNA levels of CsNBR1 and CsRHY1A in healthy (Mock) and *C*Las-infected citrus plants, determined by RT-qPCR and normalized to *CsActin*. Values are means ± SD (n = 3) (***P* < 0.01, *****P* < 0.0001, Student’s *t*-tests). (c) Immunoblot analysis of CsNBR1 protein accumulation in WT and *C*Las-infected citrus. Protein accumulation was detected with an anti-NBR1 antibody. Actin served as a loading control. The experiments were repeated three times with similar results.

**Figure S14**

**
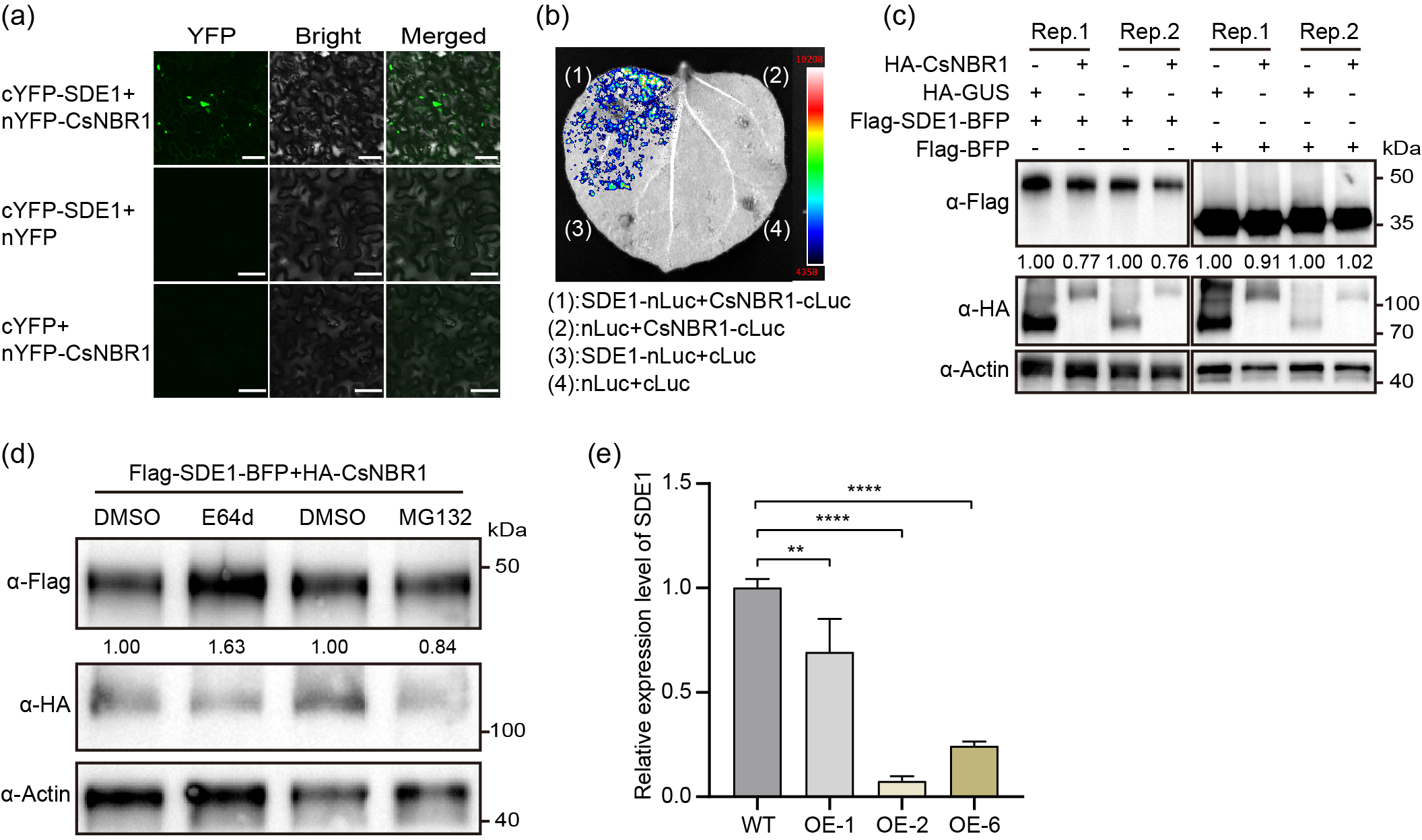
Figure S14. CsNBR1-mediated selective autophagy targets SDE1 for degradation.** **(a)** BiFC analyses of interactions between CsNBR1 and SDE1 protein. Scale bar = 50 μm. **(b)** LCI assays of CsNBR1-SDE1 interactions. SDE1-nLUC, or nLUC was co-expressed with cLUC-CsNBR1. Luciferase activity was detected at 48 hpi using a CCD. **(c, d)** CsNBR1 promotes autophagic degradation of SDE1. Immunoblot analysis of SDE1 levels in *N. benthamiana* leaves co-expressing CsNBR1 or EV. Leaves were treated with 100 μM E64d or 50 μM MG132 at 36 hpi (d), and samples were collected at 48 hpi. Protein accumulation was detected using an anti-Flag antibody. Actin was used as a loading control. **(e)** Relative mRNA levels of SDE1 in WT and *CsRHY1A*-OE citrus leaves infected with *C*Las, quantified by RT-qPCR. *CsActin* was as a control gene for normalization. Values are means ± SD (n = 3) (***P* < 0.01, ****P* < 0.001, *****P* < 0.0001, one-way ANOVA).
